# Sexual dimorphism of circadian liver transcriptome

**DOI:** 10.1016/j.isci.2024.109483

**Published:** 2024-03-12

**Authors:** Artem A. Astafev, Volha Mezhnina, Allan Poe, Peng Jiang, Roman V. Kondratov

**Affiliations:** 1 Center for Gene Regulation in Health and Disease (GRHD), Cleveland State University, Cleveland, OH 44115, USA; 2 Department of Biological Geological and Environmental Sciences, Cleveland State University, Cleveland, OH 44115, USA; 3 Center for Applied Data Analysis and Modeling (ADAM), Cleveland State University, Cleveland, OH 44115, USA; 4 Center for RNA Science and Therapeutics, School of Medicine, Case Western Reserve University, Cleveland, OH 44106, USA

**Keywords:** Nutrition in life cycle, Developmental biology, Transcriptomics, Model organism, Diet

## Abstract

Sexual dimorphism affects various aspects of physiology, metabolism and longevity. Circadian clock is a master regulator of metabolism. Anti-aging dietary interventions reprogram circadian transcriptome in the liver and other tissues, but little is known about sexual dimorphism of circadian transcriptome. We compared circadian transcriptomes in the liver of male and female mice on *ad libitum* (AL) and 30% caloric restriction (CR) diets. We found that AL female mice had a larger number of oscillating genes than male mice, and the portion of the transcriptome with sex-specific rhythms displayed phase difference. We found that CR increased the number of oscillating genes in both sexes and strongly synchronized the transcriptome without complete elimination of sex dimorphism in rhythms. Sex also had an effect on the response of the rhythms to CR. Gene ontology analysis revealed sex-specific signatures in metabolic pathways, which suggests a complex interaction of sex, circadian rhythms, and diet.

## Highlights

• Circadian liver transcriptome is sexually dimorphic
• Timed caloric restriction increases and synchronizes rhythms in gene expression
• Response of liver transcriptome to caloric restriction is sex dependent
• Sex stratification in experimental design is necessary to detect certain rhythms

## Graphical abstract

**Figure:**
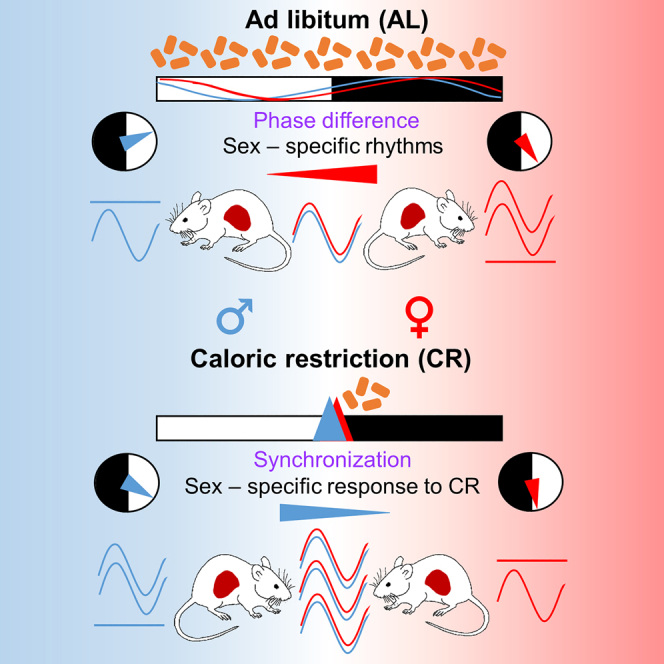

## Introduction

There is a growing body of evidence on sexual dimorphism in metabolism and physiological response to anti-aging dietary interventions like caloric restriction (CR) and time-restricted feeding.^1^^,^^2^ At the same time, there is an increasing awareness of the predominantly male-favored sex-bias across the fields in biomedical research.^3^^,^^4^ Substantial evidence suggests that sexual dimorphism exists in the liver transcriptome across species—from zebrafish to human.^5^^,^^6^^,^^7^ Various studies reported sexual dimorphism in liver gene expression—ranging from 40 to 50 genes to more than a half of liver transcriptome, depending on statistical power and magnitude of the difference.^8^^,^^9^ Since metabolism is strongly affected by circadian rhythms,^10^^,^^11^ it is essential to account for the time-dependent differences in gene expression which may originate from circadian oscillation.

Circadian clock and rhythms align metabolism with a periodic 24-h light/dark cycle in the environment.^12^^,^^13^ Robust circadian rhythms have a positive effect on health while disturbance of the rhythms causes metabolic disorders and may lead to premature aging.^14^^,^^15^^,^^16^^,^^17^^,^^18^ In mammals, the central clock is located in the suprachiasmatic nucleus (SCN) of the hypothalamus and is entrained by the periodic light/dark cycle.^19^ Peripheral clocks which orchestrate the rhythms in other tissues receive the signals from both the central clock and external cues such as feeding.^20^ On a molecular level, circadian rhythms are orchestrated by transcriptional-translational feedback loop.^21^

Though sexual dimorphism in morphology and input/output signaling was well documented for the central circadian pacemaker in the SCN,^22^^,^^23^^,^^24^ less is known on sexual dimorphism in peripheral tissues. Circadian transcriptome was found to have remarkable plasticity in response to diet, but the studies were performed either with male animals^25^^,^^26^^,^^27^^,^^28^ or with a mixed sample that included both sexes without stratification.^29^ Emerging evidence suggests the existence of sexual dimorphism on transcriptomic level from mouse to human in a variety of physiological contexts.^30^^,^^31^^,^^32^ At the same time, some circadian clock genes display sexual dimorphic expression in mouse liver on *ad libitum* (AL) or CR diet.^33^

In this study, we have performed a systematic analysis of circadian rhythms in the liver transcriptome for male and female mice on AL and CR diets. We found that gene expression rhythms and their response to the diet were sex dependent.

## Results

### Sexual dimorphism in circadian liver transcriptome under AL control conditions

**Figure 1:**
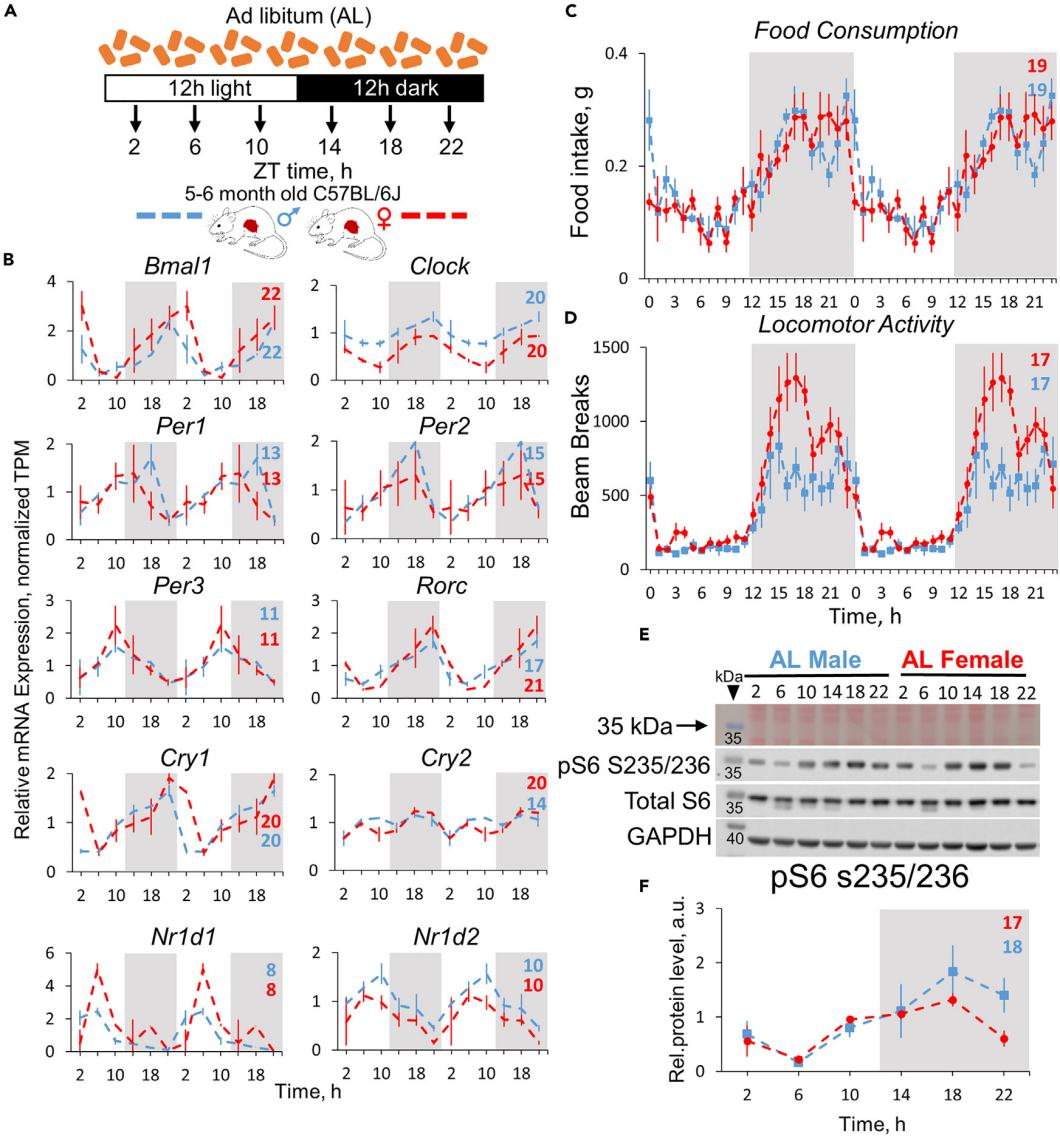
Circadian rhythms in physiology, mTOR signaling, and core clock gene expression in *ad libitum* mice (A) Experimental design: the food and water was provided to the mice in unrestricted manner. The tissues were collected every 4 h, as indicated. The light was on at ZT0 and off at ZT12. (B) Gene expression profiles of core clock genes in the liver. Data are presented as mean of normalized TPMs ± SEM, n = 3 per time point, per group; data are normalized on AL male daily average and double-plotted. Respective phases are shown as numbers on every graph (colored blue for male, red - for female mice). (C) Rhythms in food intake, measured hourly for 3 days (Mean of n = 5 ± SEM per group, per time point). (D) Locomotor activity, recorded hourly for 5 days (Mean of n = 6 ± SEM per group, per time point). For food intake and locomotion represented as % of total daily intake/locomotion, see Figure S1. (E) Representative blots showing phosphorylation of ribosomal protein S6 in mouse liver (3 pooled samples per time point, per group). (F) Quantification of the listed blot normalized on total protein and GAPDH (Average of n = 3 ± SEM per time point, per group, see Figure S1J).

First, we compared rhythms in feeding, locomotion, and metabolic signaling to see whether their rhythms are synchronized under control diet (Figure 1A). mRNA expression for 2 out of 10 core clock genes demonstrated the phase shift between sexes: *Rorc* (4-h shift) and *Cry2* (6-h shift), (Figure 1B). We observed no phase shift in feeding (Figures 1C and S1C); as expected, mice consumed majority of the food during the dark phase of the daily cycle with peaks at ZT19 for both sexes. No significant difference in total daily food intake was observed between sexes (Figure S1H). No phase shift was observed in locomotor activity (Figures 1D and S1D) between males and females; activity peaked at ZT17 in both sexes. Female mice under AL showed a trend towards increased total daily locomotor activity compared to males (2-tailed t test, p = 0.055)—the effect that was previously reported to be significant.^30^^,^^34^ Mechanistic target of rapamycin (mTOR) pathway is a sensor of nutrients and master regulator of cell metabolism in response to feeding.^35^ Phosphorylation of ribosomal protein S6 at serine 235/236 is a common marker of mTOR complex 1 activity *in vivo.*^36^^,^^37^ In line with the food intake data, S6 phosphorylation was rhythmic in both sexes with the peak at ZT17 for females and ZT18 for males (Figures 1E, 1F, and S1J).

**Figure 2:**
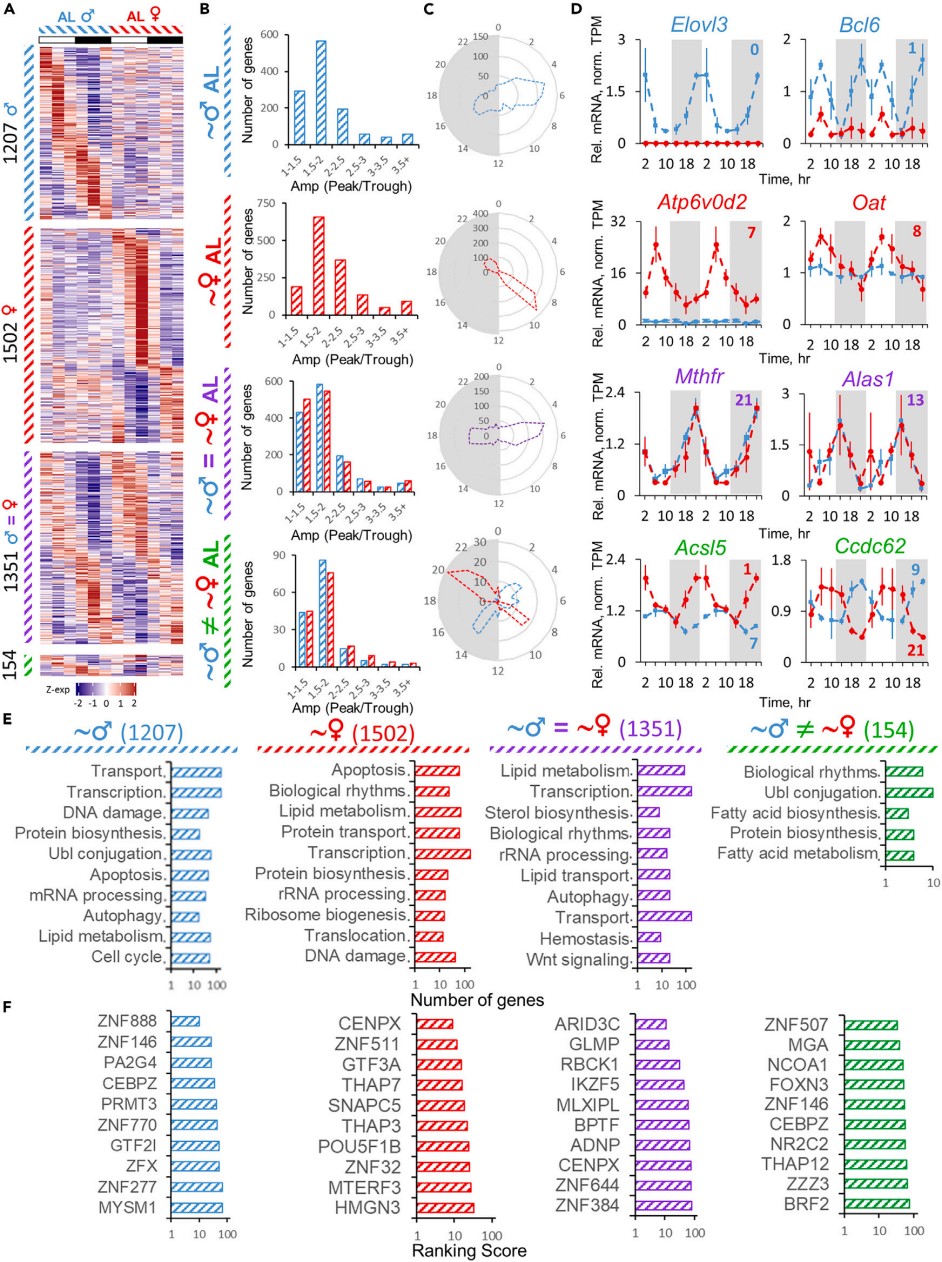
Rhythms in liver transcriptome from *ad libitum* mice (A) Heatmaps of gene expression profiles identified as differentially or similarly rhythmic between sexes under AL diet by CompareRhythms (see STAR Methods section, Table S2 and the color coding guide at the end of this legend) clustered by phase. Data are presented as *Z* scores of averages (n = 3) for each time point. (B) Histograms of amplitude distribution represented as peak/trough ratio for the respective group of genes indicated by label and color coding. (C) Radar plots of phase distribution assigned by CompareRhythms for the respective group of genes. (D) Gene candidates showing sexual dimorphism in circadian rhythm of RNA expression profile. Data are presented as average (n = 3 ± SEM per group, per time point; data are normalized on AL male daily average) of normalized TPM (transcripts per million, see Tables S1 and S2). Data are double-plotted. Respective phases are shown as numbers on every graph (colored blue for male and red for female mice). (E) Top ten enriched Biological Process (BP) terms for each of the designated groups of genes. (F) Top ten transcription factors identified by ChEA3-ChIP-X Enrichment Analysis for each of the designated groups of genes. Color coding and labels for the figure: blue dash (♂) – genes rhythmic in AL male liver; red dash (♀) – genes rhythmic in AL female liver; purple dash (♂ = ♀) – genes that are rhythmic and have the same phase in both sexes under AL diet; green dash (♂≠♀) – genes that are rhythmic in both sexes, but have a different phase in each sex under AL diet.

The comprehensive transcriptomic analysis revealed that more genes were rhythmic in female liver (Figure 2A; Tables S1 and S2) in agreement with mouse^30^ and human data.^32^ About half of the genes (50% and 45%) demonstrated rhythmic expression with the same phase in both male and female liver, respectively. 50% of genes in female and 44% in male liver showed sex-specific rhythmicity under AL, and a small fraction—154 or 5%—of the genes showed rhythmicity in both male and female liver with a phase difference between sexes (Figure 2A). As expected, genes with sexually dimorphic rhythmicity showed sex-biased amplitude distribution, while genes rhythmic in both sexes showed a similar one (Figure 2B). The phase distribution showed two major peaks around the middle of the day (when mice are least active) and in the middle of the night (when they are most active) (Figure 2C). For the genes rhythmic in both sexes we observed majority peaking at ZT5 and ZT17-18, while sex-specific rhythmic genes showed a difference in phase distribution with a roughly 4-h delay in females—ZT5 vs. ZT9 during the day and ZT16 vs. ZT21 during the night in males vs. females, respectively (Figure 2C). To a smaller extent similar distribution was recently observed in human liver.^32^ This phase difference was also retained for 154 genes that oscillated in both sexes with a phase shift between sexes (Figure 2C). Illustrative examples of the genes rhythmic only in males (*Elovl3*, *Bcl6*), only in females (*Atp6v0d2*, *Oat*), in both sexes with the same phase (*Mthfr*, *Alas1*), and in both sexes with phase difference (*Acsl5*, *Ccdc62*) are shown in Figure 2D.

Functional annotations revealed sex-specific enrichment of the genes with sex-biased oscillation in biological processes; the genes that oscillated only in males were involved in protein transport and biosynthesis, transcription, and DNA damage response (Figure 2E). The genes that oscillated in females were enriched in apoptosis, biological rhythms, lipid metabolism, and ribosomal biogenesis (Figure 2E). As expected, the genes identified as rhythmic in both sexes were involved in biological rhythms, but also in lipid metabolism, transport, sterol biosynthesis, and autophagy (Figure 2E). Accordingly, ChIP-X Enrichment Analysis 3 (ChEA3)^38^ transcription factor (TF) analysis predicted PA2G4 and MYSM1—corepressor and coactivator in androgen receptor (AR)-driven transcription, respectively^39^^,^^40^ (Figure 2F)—as enriched in male-specific rhythmic population of genes while female-specific rhythmic genes were associated with TFs involved in ribosomal biogenesis (MTERF3, GTF3A^41^^,^^42^) and regulation of transcription via histone modification (HMGN3, THAP7^43^^,^^44^) (Figure 2F). TFs predicted to regulate the genes rhythmic in both sexes included steroid coactivator NCOA1^45^^,^^46^ and MLXIPL involved in lipid storage and metabolic regulation^47^ (Figure 2F).

### CR partially synchronizes circadian transcriptome between sexes

**Figure 3:**
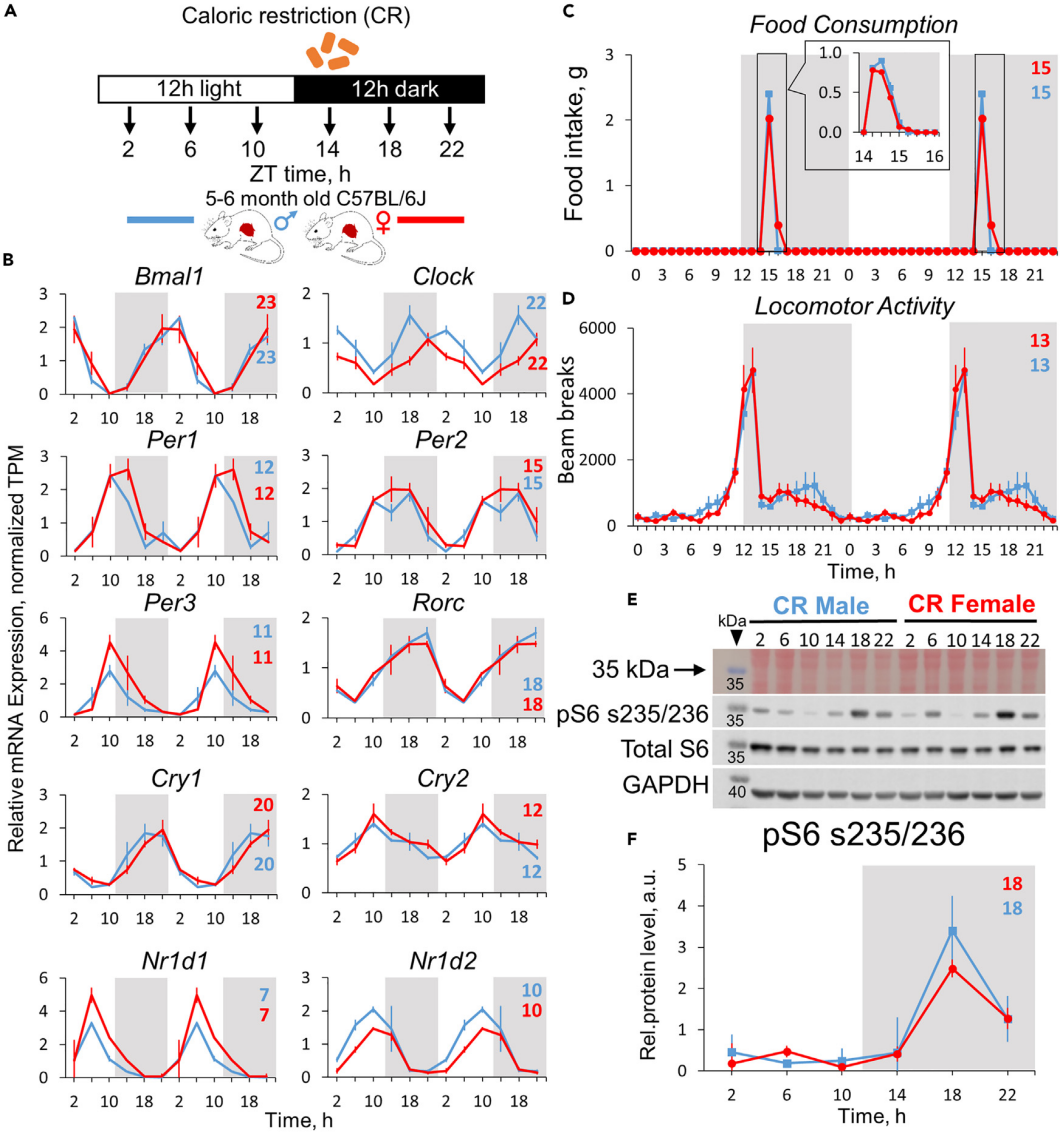
Circadian rhythms in physiology, mTOR signaling, and core clock gene expression in calorie-restricted mice (A) Experimental design: the food was provided to the mice on 30% CR at ZT14 every day, and water was not restricted. The tissues were collected every 4 h, as indicated. The light was on at ZT0 and off at ZT12. (B) Gene expression profiles of core clock genes in the liver (Data are presented as mean of normalized TPMs ± SEM, n = 3 per time point, per group; data are normalized on CR male daily average and double-plotted). Respective phases are shown as numbers on every graph (colored blue for male and red for female mice). (C) Rhythms in food intake, measured hourly + measured every 15 min for 3 days (Mean of n = 3 ± SD per group, per time point). (D) Locomotor activity, recorded hourly for 5 days (Mean of n = 7 for males and n = 5 for females ± SEM per group, per time point). For food intake and locomotion represented as % of total daily intake/locomotion, see Figure S1. (E) Representative blots showing phosphorylation of ribosomal protein S6 in mouse liver. (F) Quantification of the listed blot normalized on total protein and GAPDH (average of n = 3 ± SEM per time point, per group, See Figure S1J).

We found a difference in the rhythms between male and female liver transcriptome under AL diet. CR is known to enhance circadian rhythms in the liver.^29^ We subjected mice to 30% CR with the food provided as a single meal per day at ZT14 (Figure 3A). Since the observed phase difference in circadian liver transcriptome between males and females did not correlate with locomotion, feeding, or metabolic cycle phases under AL diet, we decided to see whether this difference can be affected by feeding intervention. After 2 months on CR the mouse bodyweight decreased by 24% for males and 14% for females (Figure S1A), and serum glucose decreased by 32% in males and 26% in females (Figure S1B). In agreement with previous data,^48^^,^^49^ CR mice consumed the food within the first 2 h (Figure 3C) and demonstrated robust locomotor activity that started about 3 h before the food was provided (Figure 3D). Total daily locomotor activity was significantly increased in both sexes under CR compared to AL condition (Figure S1I). The peaks of feeding (ZT15) and locomotor activity (ZT13) were the same in both sexes (Figures 3C, 3D, S1C, and S1D). The rhythms in RPS6 s235/236 phosphorylation were highly synchronized between sexes with a peak at ZT18 (Figures 3E, 3F, and S1J). The expression of core circadian clock genes was completely synchronized (Figure 3B). Thus, rhythms in behavior, feeding, metabolic signaling, and clock gene expression were highly synchronized under CR diet in both sexes.

**Figure 4:**
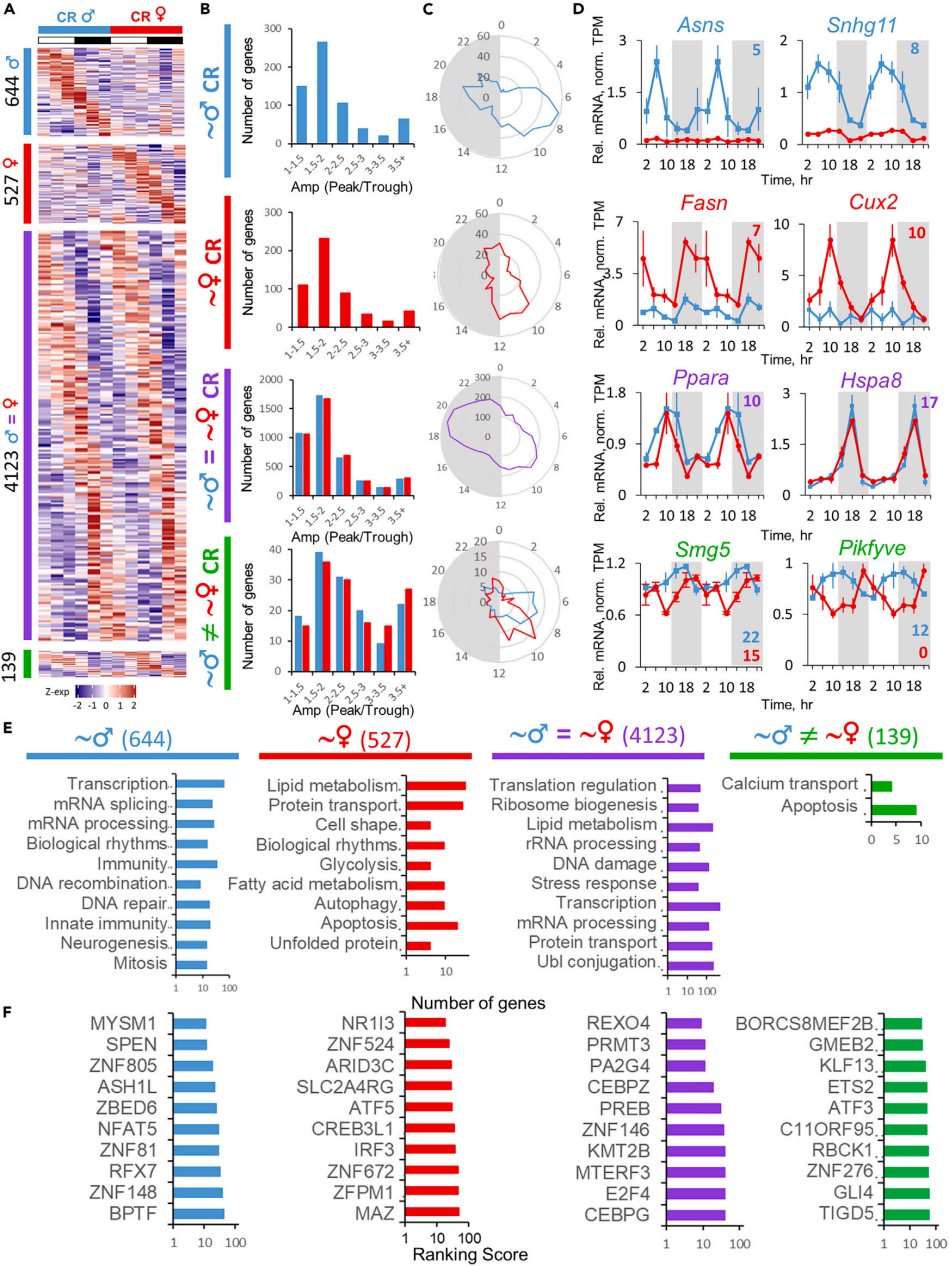
Rhythms in liver transcriptome from calorie-restricted mice (A) Heatmaps of gene expression profiles identified as differentially or similarly rhythmic between sexes under CR diet by CompareRhythms (see STAR Methods section, Table S2 and the color coding guide at the end of this legend) clustered by phase. Data are presented as *Z* scores of averages (n = 3) for each time point. (B) Histograms of amplitude distribution represented as peak/trough ratio for the respective group of genes indicated by label and color coding. (C) Radar plots of phase distribution assigned by CompareRhythms for the respective group of genes. (D) Gene candidates showing sexual dimorphism in circadian rhythm of RNA expression profile. Data are presented as average (n = 3 ± SEM per group, per time point; data are normalized on CR male daily average) of normalized TPM (transcripts per million, see Tables S1 and S2). Data are double-plotted. Respective phases are shown as numbers on every graph (colored blue for male and red for female mice). (E) Top ten enriched Biological Process (BP) terms for designated groups of genes. (F) Top ten transcription factors identified by CheA3-ChIP-X Enrichment Analysis for each of the designated groups of genes. Color coding and labels for the figure: blue solid line (♂) – genes rhythmic in CR male liver; red solid line (♀) – genes rhythmic in CR female liver; purple solid line (♂ = ♀) – genes that are rhythmic and have the same phase in both sexes under CR diet; green solid line (♂≠♀) – genes that are rhythmic in both sexes, but have a different phase in each sex under CR diet.

We hypothesized that timed feeding under CR would synchronize the oscillation of liver transcriptome between sexes. We observed that more genes were rhythmic under CR than under AL in both sexes and rhythmic transcriptome was robustly synchronized; the fractions of sex-specific rhythmic genes fell to 11% in female and 13% in male liver (Figure 4A; Tables S1 and S2). The fraction of the genes rhythmic in both sexes with phase difference decreased to 2.9% and 2.8% in female and male liver, respectively. The phase distribution for these genes was also more synchronized (Figure 4C). The fractions of genes that showed sex-specific rhythm under CR still showed 2–3 h phase difference in peak distribution between sexes, and overall CR shifted the phase distribution for all categories of genes compared to AL by 2–3 h (Figures 2C and 4C). Examples of genes oscillating only in males (*Asns*, *Snhg11*), only in females (*Fasn*, *Cux2*), and in both sexes with the same (*Ppara*, *Hspa8*) and different (*Smg5*, *Pikfyve*) phases are shown in Figure 4D.

Functional signatures of the genes with male-specific oscillation under CR were involved in transcription and RNA processing, as well as immune response and DNA repair (Figure 4E). TFs that were predicted for this group of genes included coactivator of androgen receptor (AR) MYSM1^40^ which seems to have predicted target genes among male-rhythmic gene population irrespective of the diet, histone N-methyltransferase ASH1L,^50^ Igf2—repressor ZBED6^51^ and NFAT5 involved in regulation of immune responses^52^ (Figure 4F). The genes that were rhythmic specifically in female liver showed association with fatty acid metabolism, protein transport, glycolysis, autophagy, and unfolded protein response (Figure 4E). Among the top predicted TFs for this group were NR1I3, involved in xenobiotic detoxification, energy homeostasis, and lipid metabolism,^53^ regulator of proteostasis CREB3L1,^54^ IRF3, involved in inflammation and glucose homeostasis,^55^ and MAZ—transcriptional activator binding promoters of serotonin receptor c-myc and insulin^56^ (Figure 4F). The population of genes that oscillated in both sexes under CR was associated with translation regulation, ribosome biogenesis, rRNA processing, protein transport, stress response, and Ubl-conjugation pathway (Figure 4E). TFs that were predicted in this group of genes were RNA exonuclease REXO4, MTERF3, involved in mitochondrial ribosomal biogenesis,^41^ PREB, essential in COPII-mediated endoplasmic reticulum (ER) traffic,^57^^,^^58^ CAAT-enhancer binding CEBPZ and CEBPG, as well as H3K4 histone methyltransferase KMT2B (Figure 4F). Interestingly, a small fraction of commonly rhythmic genes with phase difference between sexes was enriched in genes involved in calcium transport and apoptosis (Figure 4E); the TFs predicted for this group of genes included cyclic AMP-dependent ATF3 and glucocorticoid modulatory element binding GMEB2. Glucocorticoid-dependent cyclic AMP-mediated Ca2+ increase was previously reported in promotion of apoptosis^59^ while GMEB2 showed anti-apoptotic property.^60^

### Response of liver transcriptome to CR is sex dependent

**Figure 5:**
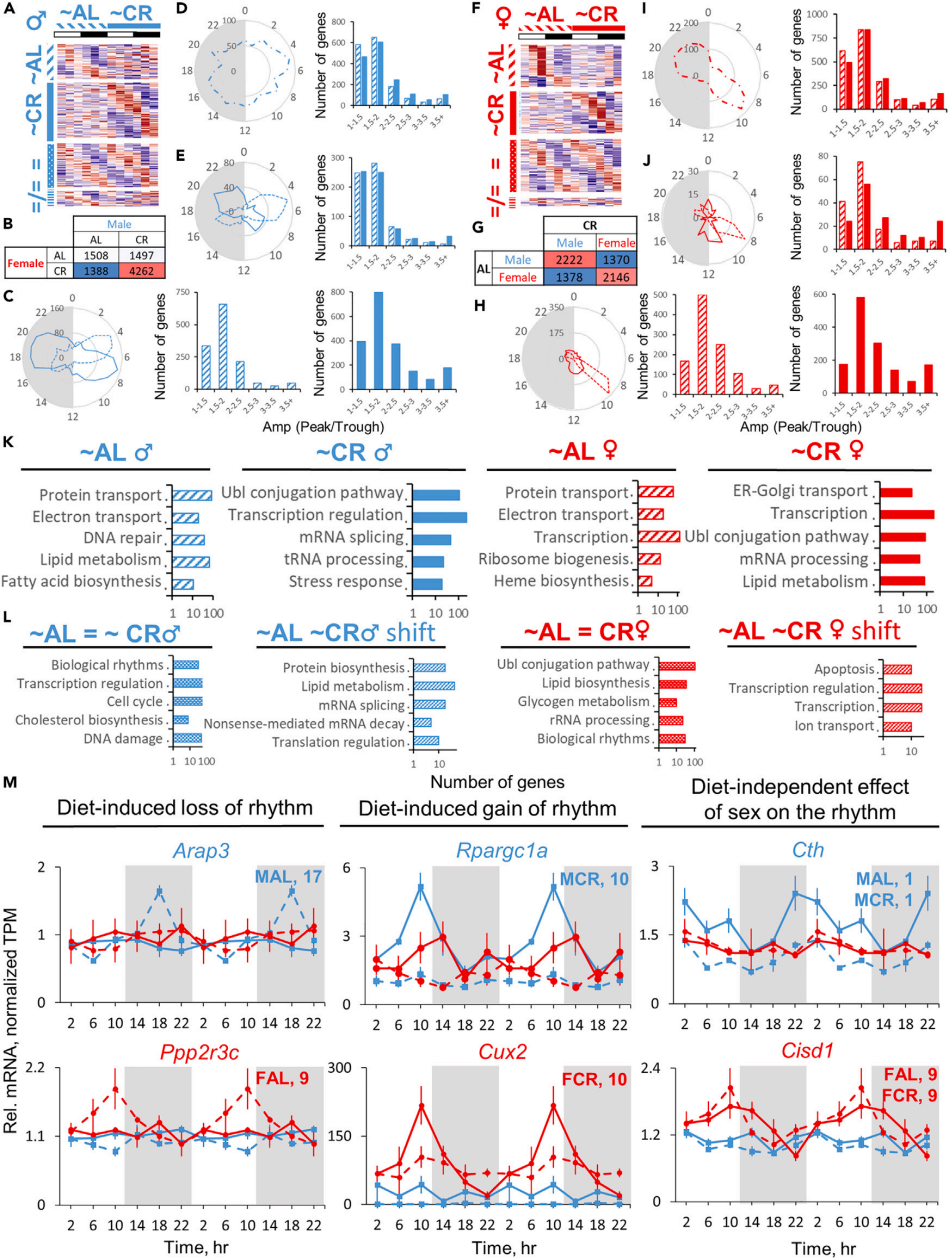
Changes in circadian rhythms of gene expression in response to caloric restriction with sex as a factor (A and F) Heatmaps of gene expression profiles identified as differentially or similarly rhythmic between diets by two independent pairwise CompareRhythms comparisons (AL male vs. CR male; AL female vs. CR female, see Tables S1 and S2). Genes were clustered by phase. Data are presented as *Z* scores of averages of normalized TPMs (n = 3) for each time point. (B and G) Matrix of the overlaps of rhythmic genes between sexes in each diet (B) and between diets in each sex (G)—done using Venn diagram method on the results of two pairwise AL vs. CR comparisons. No additional statistical analysis was used to build matrices. (C–E) Radar plots of the phase distribution and matching histograms of amplitude distribution in the groups of genes in male liver: (C) diet-specific rhythmic genes; (D) rhythmic under both diets with the same phase; (E) rhythmic under both diets with a phase shift between diets. (H–J) Same parameters (phase and amplitude distribution) in female liver: (H) diet-specific rhythmic genes; (I) rhythmic under both diets with the same phase; (J) rhythmic under both diets with a phase shift between diets. The amplitude parameter was calculated as a ratio of peak to trough. (K and L) Top five enriched Biological Processes (BP) for the groups of genes: with diet-specific rhythms (K) and diet-independent rhythms (L) (as per color coding). (M) Candidate genes showing sex-specific effects on circadian rhythms in liver gene expression in response to CR (Mean of normalized TPMs, n = 3 ± SEM per group, per time point; data are normalized on AL male daily average and double-plotted). Four pairwise CompareRhythms analyses were conducted to confirm sex and diet effects on rhythmicity in panel M (see STAR Methods, Table S2). Color coding for the figure: blue color for male liver, red for female; dashed lines/bars – genes rhythmic specifically under AL; solid lines/bars – genes rhythmic specifically under CR; equal sign (=) – genes that retain rhythm and the same phase under both diets; “not equal” symbol (≠) – genes that retain rhythm under both diets, but with a shift in the phase between diets.

CR triggers changes in gene expression rhythms.^33^^,^^61^ While CR increases the amount of rhythmic genes in both sexes, we hypothesized that some rhythmic genes under AL might display arrhythmic oscillation under CR, and vice versa. Indeed, we found that 37% of the genes rhythmic under AL in male liver lost rhythmicity under CR, and 47% of the genes gained oscillation under CR (Figure 5A). For female liver it was 33% and 40%, respectively, meaning a slightly larger fraction of genes in female liver oscillates independently from the diet (Figure 5F). We also noted that a larger fraction of diet-independent rhythmic genes in male liver had a phase difference between diets—28% vs. 8% in female liver (Figures 5A and 5F). This shows a remarkable plasticity and dependence of the transcriptomic oscillation on the type of feeding regimen (Figures 5B and 5G). Indeed, only a half of rhythmic genes oscillate independently from the diet in male liver, while 2/3 do so in female one. Additionally, CR shifts the phase distribution in both sexes (Figures 5C and 5H); majority of peaks are delayed by 2–3 h compared to AL (Figure 2C) even for the population of genes that are rhythmic independently of the diet (Figures 5E and 5J). In order to better illustrate these findings, we have subdivided the genes into three categories with representative examples: diet-induced loss of rhythms: *Arap3* in males and *Ppp2r3c* in females; diet-induced gain of rhythms: *Ppargc1a* in males and *Cux2* in females; and diet-independent effect of sex on the rhythms: *Cth* in males and *Cisd1* in females (Figure 5M). Functional annotation showed that CR reprogrammed biological processes of rhythmic genes protein transport, DNA repair and lipid metabolism to Ubl conjugation pathway, mRNA splicing, and stress response in male liver (Figure 5K). In female liver protein transport, ribosome biogenesis and heme biosynthesis were reprogrammed to ER-Golgi transport, transcription, Ubl conjugation pathway, and lipid metabolism (Figure 5K). Biological rhythms were enriched in both sexes among diet-independent rhythmic genes, while cholesterol biosynthesis was enriched in males and glycogen metabolism in females (Figure 5L). These data show that in both sexes diet is a strong determinant of the rhythmic transcriptional programs highlighting the importance of sex in response to CR.

### Sex stratification in experimental design is necessary to identify certain gene oscillations

**Figure 6:**
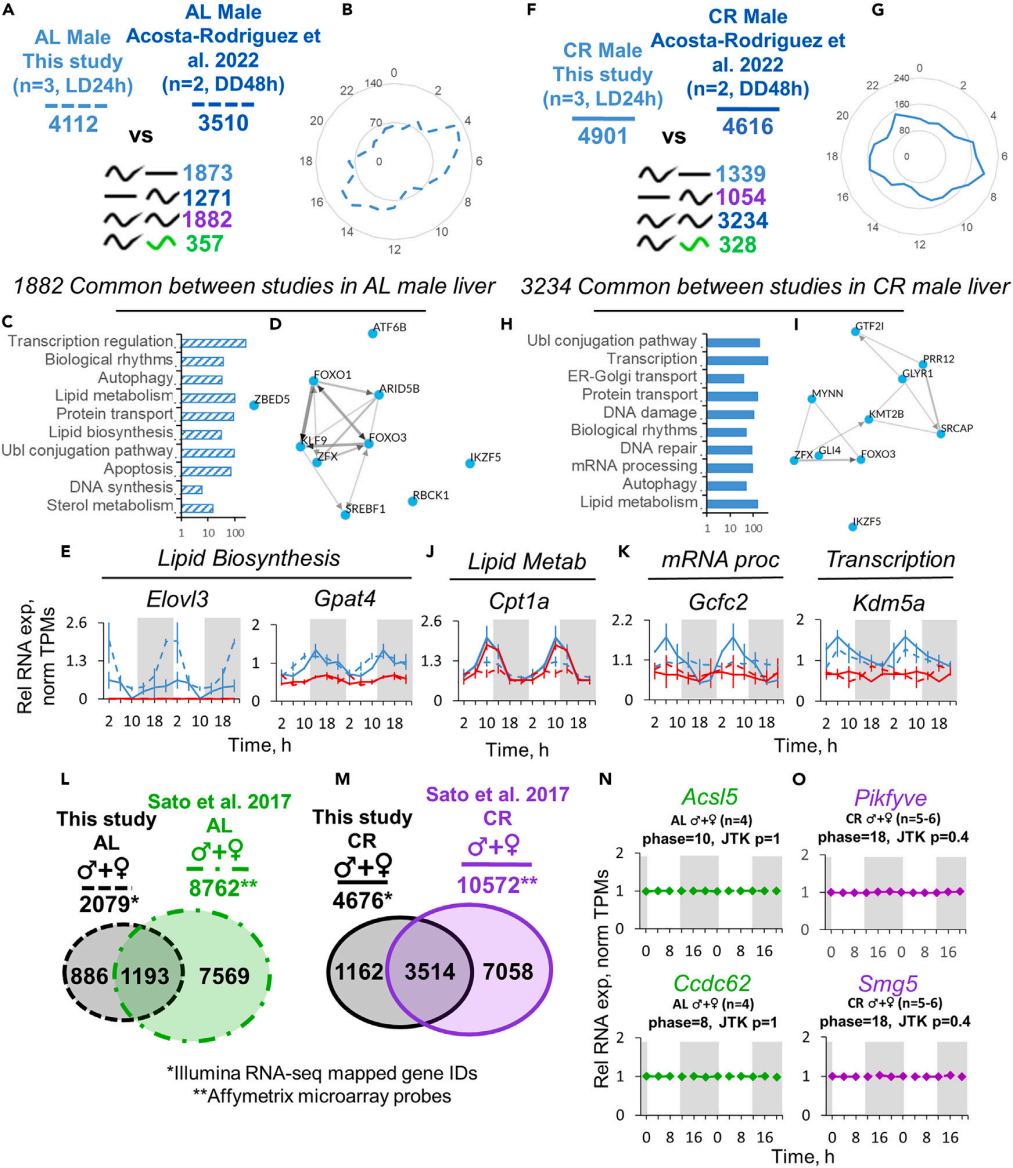
Effects of data sex stratification, diet, and data source on detection of oscillating genes (A and F) Genes with similar and differential rhythmicity assessed by two direct pairwise CompareRhythms comparisons (AL vs. AL, CR vs. CR; per = 24 h, BICw Schwartz>0.4, normTPM>1) between this study (Light-Dark condition, n = 3 per time point, 6 time points over 24 h) vs. Acosta – Rodriguez et al. 2022^48^ (GEO: GSE190939) (Constant Darkness, n = 2 per time point, 12 time points over 48 h) under each respective diet. Raw RNA-seq data from both studies were processed as described in STAR Methods before analysis. Dashed lines represent *ad libitum*, solid lines represent caloric restriction. (B and G) Radar plots of the genes identified as rhythmic by both studies under AL (B) and CR (G). (C and H) Top enriched biological process (BP) signatures of the genes identified as rhythmic by both studies under AL (C) and CR (H). (D and I) Network of the top CheA3-predicted transcription factors enriched in the group of genes rhythmic in both studies under AL (D) and CR (I). (E and K) RNA-seq gene expression profiles of the sexually dimorphic rhythmic gene candidates from characteristic BP terms. Data are presented as Mean of normalized TPMs ± SEM (n = 3, LD, 24 h, 6 time points double plotted). (J) Example of sex- and diet-independent rhythmicity in expression. Data are presented as Mean of normalized TPMs ± SEM (n = 3, LD, 24 h, 6 time points double plotted). Blue lines represent data from male liver samples, red – from female. Dashed lines represent AL diet. Solid lines represent CR diet. Four pairwise CompareRhythms analyses were conducted to confirm sex and diet effects on rhythmicity in (E), (K), and (J) (see STAR Methods, Table S2). (L and M) Venn diagrams representing independently identified rhythmic genes from this analysis (RNA-seq) and Sato et al. 2017^29^ (Microarray; GEO: GSE93903) (JTK, p < 0.05; Light-Dark conditions, no pairwise statistical analysis was done to build Venn Diagram). Data are presented as mean of n = 4–6, both male and female liver samples averaged together. Dashed lines represent AL diet, solid lines – CR diet. Black color represents this study, green – AL samples from Sato et al. 2017, purple – CR samples from Sato et al. 2017. (N and O) Representative candidate genes plotted from Sato et al. 2017 microarray data (Mean ± SEM, n = 4–5 for AL; n = 5–6 for CR). Green – AL, purple – CR. Data are showing that using the pooled male + female sample these genes are not identified as rhythmic – in agreement with the data from our analysis presented in the main text Figures 2D and 4D (see Tables S1 and S2).

In order to benchmark our study and assess the importance of sex as a factor in detection of mRNA rhythmicity, we have compared our data with existing reports on the effect of CR on liver circadian rhythms^28^^,^^29^ and effect of microbiota depletion on sex-specific rhythms in gene expression.^30^ Direct comparison of our and Acosta-Rodriguez et al.^28^ data from male liver samples revealed a 46% match of rhythmic genes under AL and 66% under CR; these fractions of genes also had matching phases between reports (Figures 6A, 6B, 6F, and 6G). The phase distributions of the matching rhythmic genes were similar to the ones reported by us in Figures 2C and 4C. Though in different order, similar functional annotations were identified (Figures 6C and 6H). The respective TFs predicted for this group of genes are provided in Figures 6D and 6I. Importantly, among the genes under respective functional annotations, we found the genes with sex-specific rhythmicity under both AL (Figure 6E) and CR conditions (Figure 6K). While the studies performed on exclusively male samples (such as Acosta-Rodriguez et al.^28^) may provide information about oscillations in the genes like *Cpt1a* (that matched between our studies and applied to both sexes, Figure 6J), this might not be the case for the genes with diet-dependent (*Elovl3*, *Gcfc2*) and diet-independent (*Gpat4*, *Kdm5a*) sexual dimorphism in rhythmicity (Figures 6E and 6K).

Some studies may use statistical sample of mixed sex, as was the case in Sato et al.’s report.^29^ For this comparison we pooled male and female samples in our data and evaluated the overlap of independently identified rhythmic genes. Even though platform differences did not allow us to compare the data directly, we have observed 57% of our rhythmic genes match Sato et al. under AL and 75% under CR (Figures 6L and 6M). While this result may be prone to bias from RNA sequencing (RNA-seq) vs. microarray comparison, it points to the finding that essentially under any experimental setup more rhythmic genes are detectable under CR in the liver. However, what mixed-sex approach will not be able to identify are the rare genes that are rhythmic in both sexes, but with a large phase difference (Figures 6N and 6O). These genes are efficiently identified by CompareRhythms analysis (Figures 2D and 4D), provided sex stratification is accounted for in the experimental setup.

**Figure 7:**
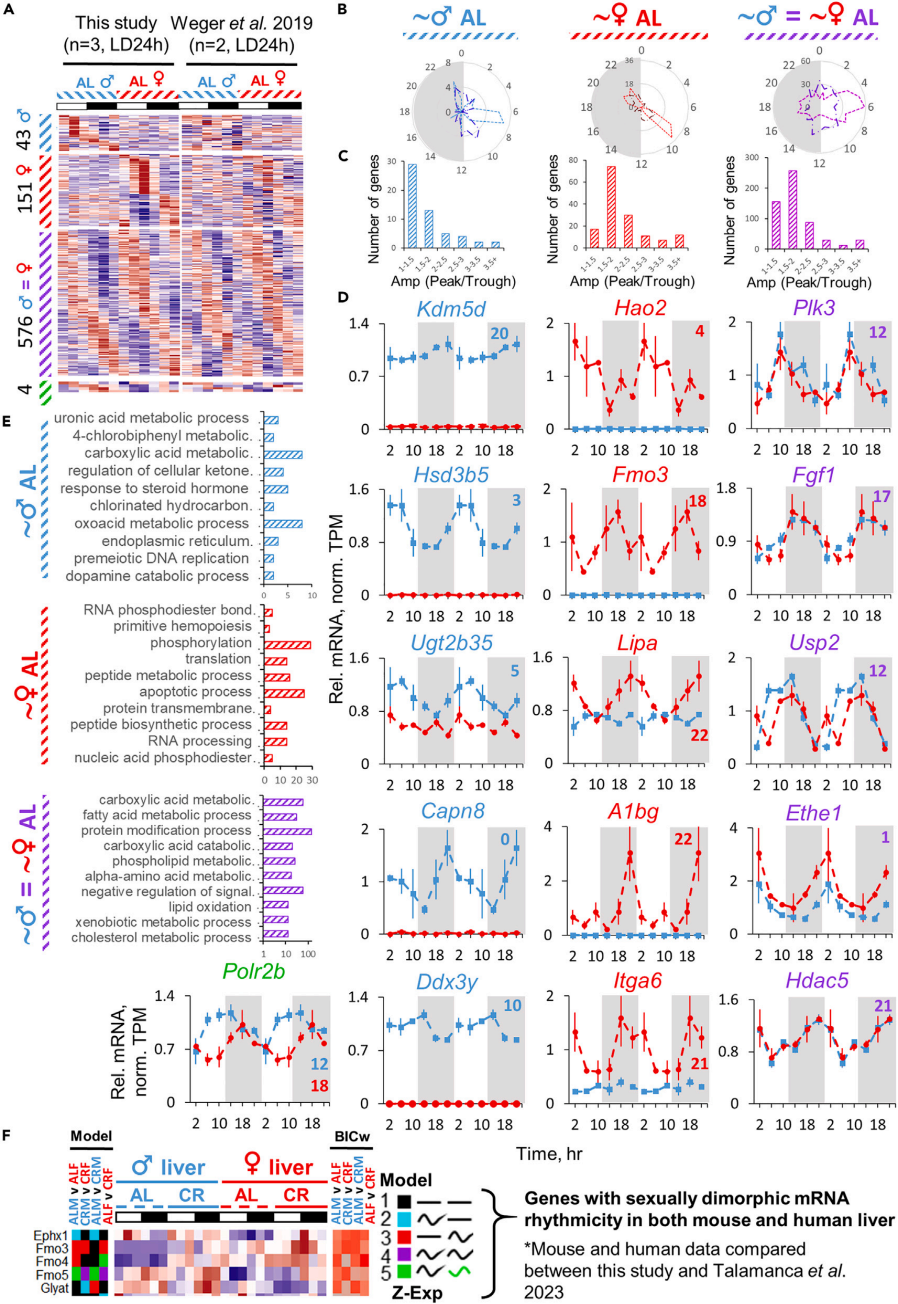
Between-report consistency of sex dimorphism in rhythmic transcriptome under AL diet (A) Heatmaps of gene expression profiles independently identified as differentially or similarly rhythmic between sexes in AL C57BL/6J mouse liver by both this study and Weger et al. 2019^30^ (RNA-seq; GEO: GSE77221) by model selection approach (per = 24h, BIC, Schwartz weight cutoff >0.4) clustered by phase (see STAR Methods section and the color coding guide at the end of this legend). Both reports used the same criteria, but only independently obtained results were overlapped for (A) using Venn diagram approach without raw RNA-seq data reprocessing (see STAR Methods). No direct statistical analysis was done for pairwise comparison between this and Weger et al. 2019^30^ data. Data are presented as *Z* scores of averages (n = 3) for each time point for this study and n = 2 for Weger et al. 2019. Conditions were 24 h (12/12 LD) for this study and for Weger et al. 2019 (see Figure S2; Tables S1 and S2). (B) Radar plots of phase distribution of sex-specific rhythmic genes and genes rhythmic in both sexes (blue - male rhythmic genes, red female - rhythmic genes; purple - genes rhythmic in both sexes; light shade - this study, dark shade - Weger et al.^30^). (C) Histograms of amplitude distribution represented as peak/trough for the respective group of genes indicated by label and color coding. The amplitude parameter was calculated as a ratio of peak to trough. (D) Candidate genes demonstrating male-specific, female-specific and sex-independent rhythmicity under AL condition identified independently by both studies. Data are presented as Mean of normalized TPMs ± SEM (n = 3, LD, 24h, 6 time points double plotted). (E) Top enriched Gene Ontology (GO) terms for male-specific, female-specific, and commonly rhythmic genes identified independently by both studies. (F) Candidate genes with sexually dimorphic rhythmic expression identified independently by this study in mice and Talamanca et al. 2023 in humans^32^ (Database: GTEx V8 (dbGaP Accession phs000424.v8.p2). Independently derived results were selected and genes with similar expression profiles were displayed. No statistical analysis was performed between raw datasets of this study and Talamanca et al. 2023.^32^

Around-the-clock sex-stratified transcriptome was recently published in the report studying microbiota depletion in mice.^30^ We have compared populations of genes identified as sexually dimorphic and non-dimorphic in rhythmicity under AL. Data were processed and analyzed independently, though a similar model-selection approach was used (refer to STAR Methods). Regardless of the batch effect, which potentially explains minor overlap between studies (Figure 7A) and differences in phase and amplitude distribution (Figures 7B and 7C), both reports independently identified prominent sex-dimorphic and sex-independent rhythmic genes (Figures 7D and S2). Gene ontology of the genes identified by both studies as male rhythmic showed enrichment in uronic acid metabolism, regulation of ketogenesis, response to steroid hormone, and ER traffic. Female-rhythmic genes identified by both studies were enriched in primitive hematopoiesis, translation, RNA processing, and apoptosis, while protein modification, alpha-amino acid metabolism, lipid oxidation, and xenobiotic and cholesterol metabolism were enriched among the genes rhythmic in both sexes. We also found consistency in female-predominant rhythmicity of mRNAs of flavin monooxygenases under AL in comparison against human data,^32^ while *Ephx1* and *Glyat* were rather rhythmic in male liver, depending on the diet in mice (Figure 7F).

### CR partially feminizes liver rhythmic transcriptome

**Figure 8:**
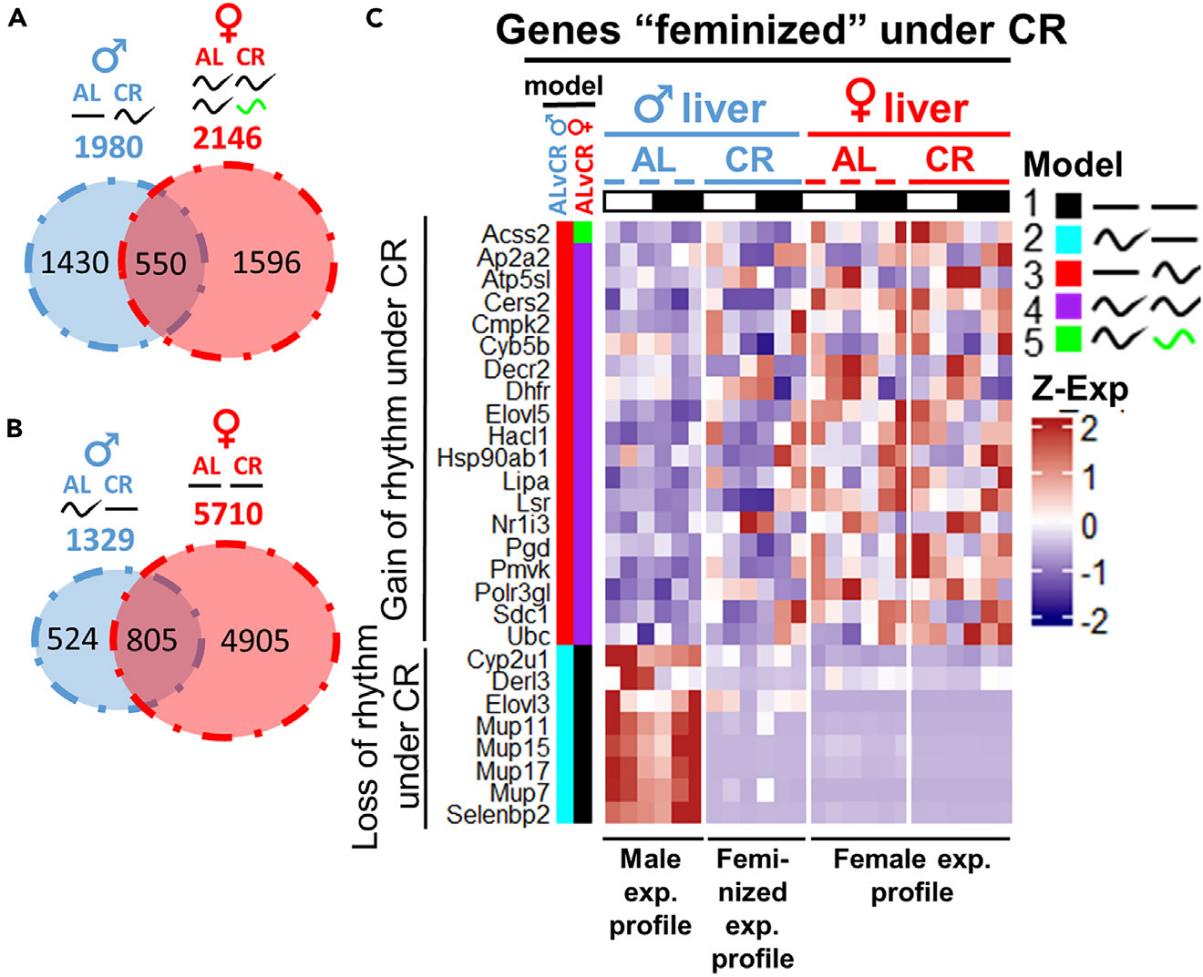
Assessment of feminization of gene expression profile under CR in male liver (A) Venn diagram of the genes that gained rhythmicity under CR in male liver against the genes that are rhythmic in female liver under both diets. (B) Venn diagram of the genes that lost rhythmicity under CR against the genes that are arrhythmic in female liver under both diets. Venn diagrams were obtained by the overlap of the results from two pairwise CompareRhythms comparisons (Al vs. CR male; AL vs. CR female, see STAR Methods, Table S2). No additional statistical analysis was applied to build Venn diagrams. (C) Heatmap with the candidate genes representing the feminizing effect of CR on male liver. Annotation on the left side of the heatmap represents the model (category) to which the genes were assigned by CompareRhythms analysis in each indicated pairwise comparison (see STAR Methods, Tables S1 and S2). Data are represented as mean of *Z* scores (n = 3) per group, per time point. Light-dark bars on top represent light and dark phase of the day. Figure color coding: Dash lines represent AL, solid lines – CR, blue – male, red – female liver.

Feminization of liver mRNA expression in response to CR has been previously reported.^62^ We decided to see whether feminization model can explain the changes in gene oscillation in male liver in response to CR. We found that about a quarter of genes that gained rhythmicity in males under CR matched female-rhythmic expression (Figure 8A), while 60% of genes that lost rhythm under CR in male liver matched female arrhythmic expression profiles, though this may be a biased estimation due to a larger arrhythmic gene population (Figure 8B). A handful of genes encoding enzymes in variety of biochemical pathways and major urinary proteins indeed follow feminization paradigm (Figure 8C), but the size of the affected gene fraction does not let us consider this effect to be systemic. Prominent gene candidates also do not demonstrate feminization of the rhythms in response to CR: *Ppp2r3c*, *Ppargc1a*, *Cux2*, *Cth*, and *Cisd1* (Figure 5M). Therefore, feminization model cannot completely explain the observed sex differences.

### Sexual dimorphism in rhythmic liver gene expression has a pathway-specific effect

**Figure 9:**
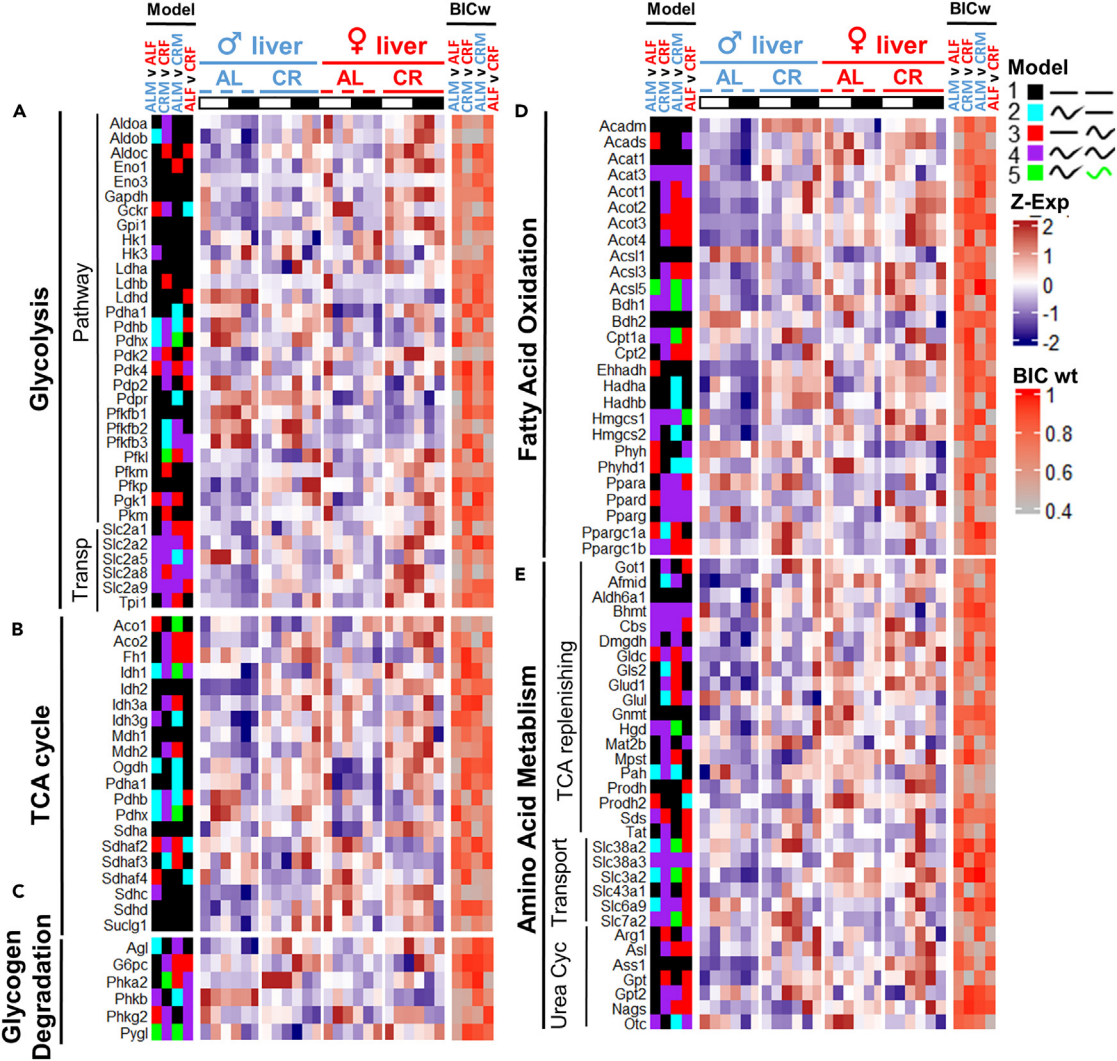
Effect of sex and diet on rhythmicity in mRNA profiles of energy producing and catabolic pathway enzymes Heatmaps of RNA-seq expression profiles of the genes involved in (A) Glycolysis, (B) TCA Cycle, (C) Glycogen degradation, (D) Fatty acid oxidation, and (E) Amino acid metabolism. For Ox-Phos genes see Figure S3, Table S1. Data are represented as mean of *Z* scores (n = 3) per group, per time point. Annotation on the left side of the heatmap represents the model (category) to which the genes were assigned by CompareRhythms analysis using four pairwise comparisons, indicated on the top of the heatmap (AL male vs. AL female; CR male vs. CR female; AL male vs. CR female; AL female vs. CR female, see Table S2). Model legend and color code is on the top right of the figure panel. Annotation on the right side of the heatmap represents BIC Schwartz weight value for each of the comparisons (see STAR Methods). Light-dark bars on top represent light and dark phase of the day. Figure color coding: Dash lines represent AL, solid lines – CR, blue – male, red – female liver. Order of the gene expression *Z* score averages within each group on the heatmap follows the time point order of the experimental design – ZT2,6,10,14,18,22. Order of the experimental groups on heatmaps is *ad libitum* male liver (♂AL); calorie-restricted male liver (♂CR); *ad libitum* female liver (♀AL); calorie-restricted female liver (♀CR) left to right, respectively.

Since circadian clock and feeding affect the expression of a handful of metabolic genes,^10^^,^^49^^,^^63^ we have decided to profile the genes of major metabolic pathways for the presence of sexual dimorphism and rhythmicity in expression. Overall, CR predominantly synchronized degradation and energy producing pathways (Figure 9). Some sex specificity was observed under AL condition, where *Aldob*, *Pdhb*, *Pdhx*, *Idh1*, *Ogdh*, *Agl*, *Pah* (Figures 9A–9C and 9E) and several amino acid transporters *Slc38a2*, *Slc3a2*, and *Slc6a9* (Figure 9E) were rhythmic in male liver, while *Gckr*, *Pdk4*, *Pgk1*, *Aco1*, *Sdha2&4*, *Phkg2*, *Acads*, *Ehhadh*, *Phyh*, *Phyhd1*, *Ppard*, *Ppargc1a*, *Gldc*, and *Prodh2* as well as some genes encoding oxidative phosphorylation components were female rhythmic (Figures 9 and S3). Diet-independent rhythmic genes were *Hmgcs1*, *Bhmt*, *Cbs*, *Acat3*, and *Slc38a3*, while *Acots* demonstrated gain of rhythmicity under CR in both sexes (Figures 9D and 9E).

**Figure 10:**
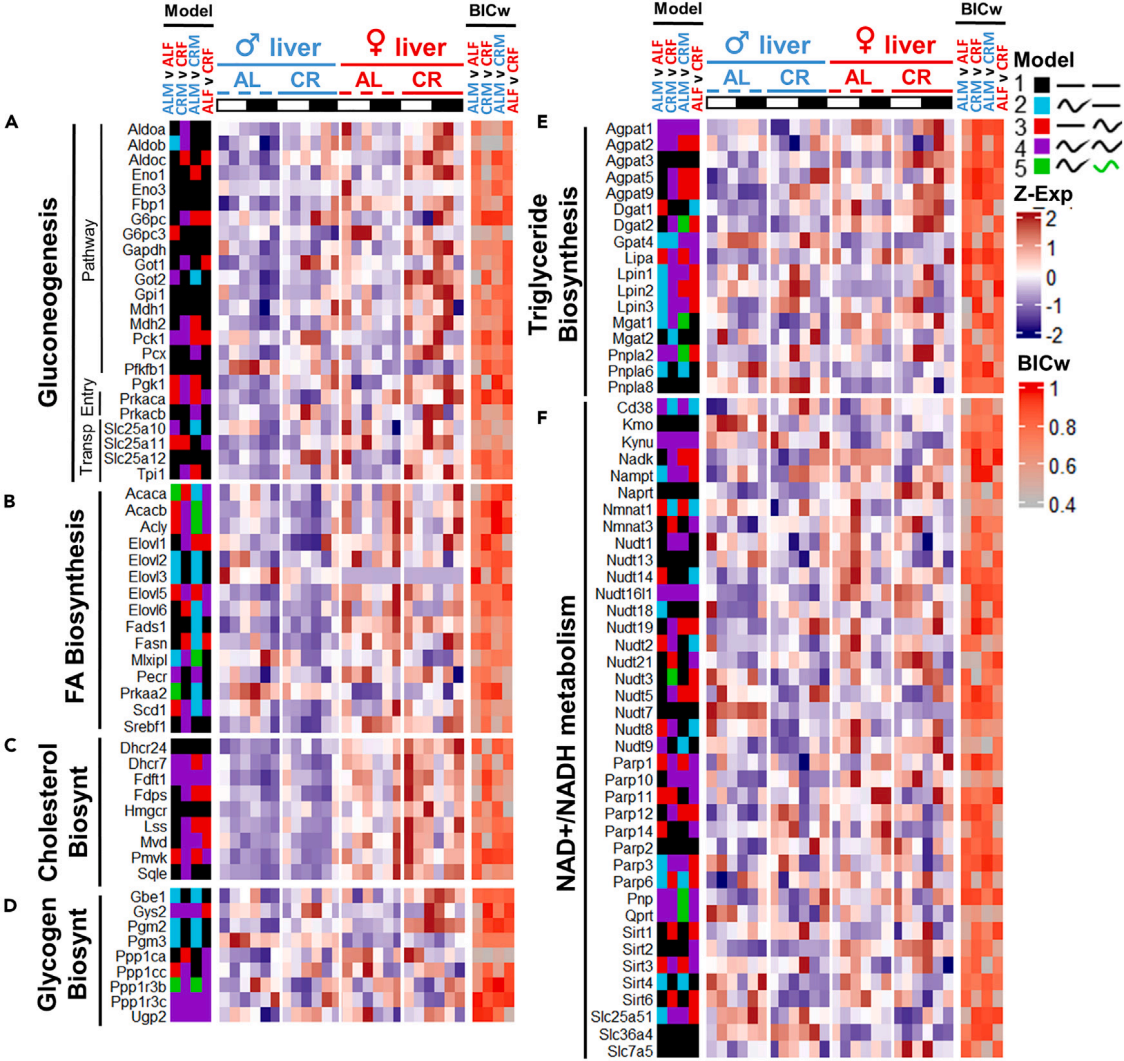
mRNA profiles of the genes with predominantly sex-independent rhythmic response to CR Heatmaps of RNA-seq expression profiles of the genes involved in (A) Gluconeogenesis, (B) Fatty acid Biosynthesis, (C) Cholesterol Biosynthesis, (D) Glycogen Biosynthesis, (E) Triglyceride Biosynthesis, and (F) NAD+/NADH metabolism. Data are represented as mean of *Z* scores (n = 3) per group per time point. Annotation on the left side of the heatmap represents the model (category) to which the genes were assigned by CompareRhythms analysis using four pairwise comparisons, indicated on the top of the heatmap (AL male vs. AL female; CR male vs. CR female; AL male vs. CR female; AL female vs. CR female, see Table S1 and S2). Model legend and color code is on the top right of the figure panel. Annotation on the right side of the heatmap represents BIC Schwartz weight value for each of the comparisons (see STAR Methods). Light-dark bars on top represent light and dark phase of the day. Figure color coding: Dash lines represent AL, solid lines – CR, blue – male, red – female liver. Order of the gene expression *Z* score averages within each group on the heatmap follows the time point order of the experimental design – ZT2,6,10,14,18,22. Order of the experimental groups on heatmaps is *ad libitum* male liver (♂AL); calorie-restricted male liver (♂CR); *ad libitum* female liver (♀AL); calorie-restricted female liver (♀CR) left to right respectively.

Biosynthetic pathways showed a slightly different distribution; genes rhythmic in gluconeogenesis—*G6pc3*, *Pgk1*, *Prkaca*, and *Slc25a11*—showed female-predominant rhythmicity under AL while most of gluconeogenetic genes were rhythmic under CR in both sexes (Figure 10A). In fatty acid biosynthesis *Scd1*, *Acacb*, *Acly*, and *Elovl5* were rhythmic in females under AL and synchronized with males under CR, while *Elovl2* and *3* were only rhythmic in AL male liver (Figure 10B). Glycogen and triglyceride biosynthesis showed male-dominant rhythmicity in males in *Gbe*, *Pgm2,3*, and *Lpin1,2,3* enzymes (Figures 10D and 10E). Genes involved in NAD+ metabolism showed sex-specific rhythms in *Sirt3,4* and several *Parps* under AL, while under CR rhythmicity was increased for both sexes—similarly to the behavior of the genes in cholesterol biosynthesis, suggesting sex is not an essential factor in response to CR in these pathways (Figures 10C and 10F).

### Endocrine control of sex differences in transcriptional oscillation

**Figure 11:**
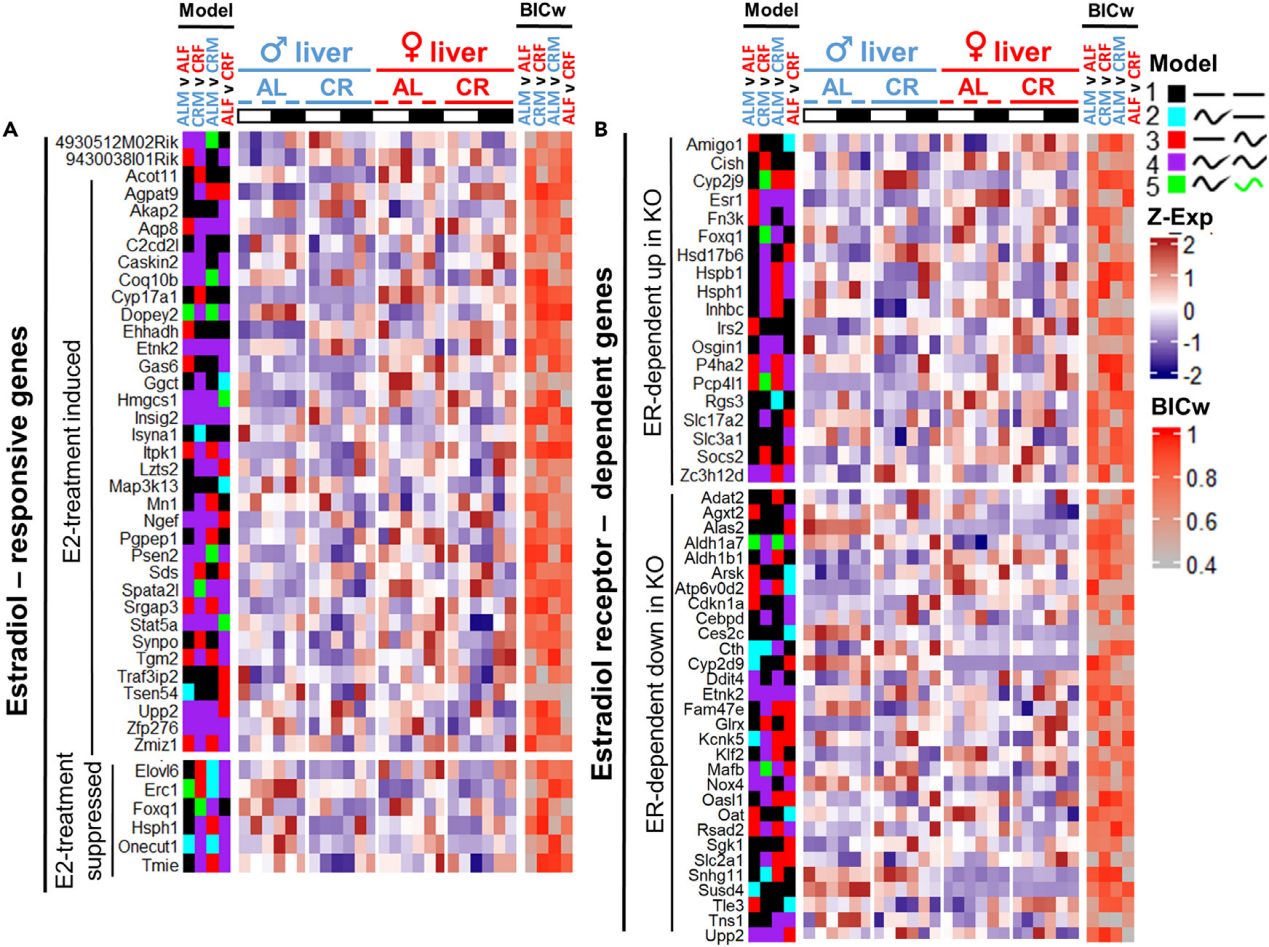
mRNA profiles of Estradiol-regulated genes The heatmaps show the genes with rhythmic expression profiles in at least one experimental group. (A) Responsive to Estradiol injection. (B) Estradiol Receptor-dependent, differentially expressed in WT vs. KO. Full gene list was imported from Supplemental information of Palierne et al. 2016.^65^ Data are represented as mean of *Z* scores (n = 3) per group per time point. Annotation on the left side of the heatmap represents the model (category) to which the genes were assigned by CompareRhythms analysis using four pairwise comparisons, indicated on the top of the heatmap (AL male vs. AL female; CR male vs. CR female; AL male vs. CR female; AL female vs. CR female, see Tables S1 and S2). Model legend and color code is on the top right of the figure panel. Annotation on the right side of the heatmap represents BIC Schwartz weight value for each of the comparisons (see STAR Methods). Light-dark bars on top represent light and dark phase of the day. Figure color coding: Dash lines represent AL, solid lines – CR, blue – male, red – female liver. Order of the gene expression *Z* score averages within each group on the heatmap follows the time point order of the experimental design – ZT2,6,10,14,18,22. Order of the experimental groups on heatmaps is *ad libitum* male liver (♂AL); calorie-restricted male liver (♂CR); *ad libitum* female liver (♀AL); calorie-restricted female liver (♀CR) left to right, respectively.

**Figure 12:**
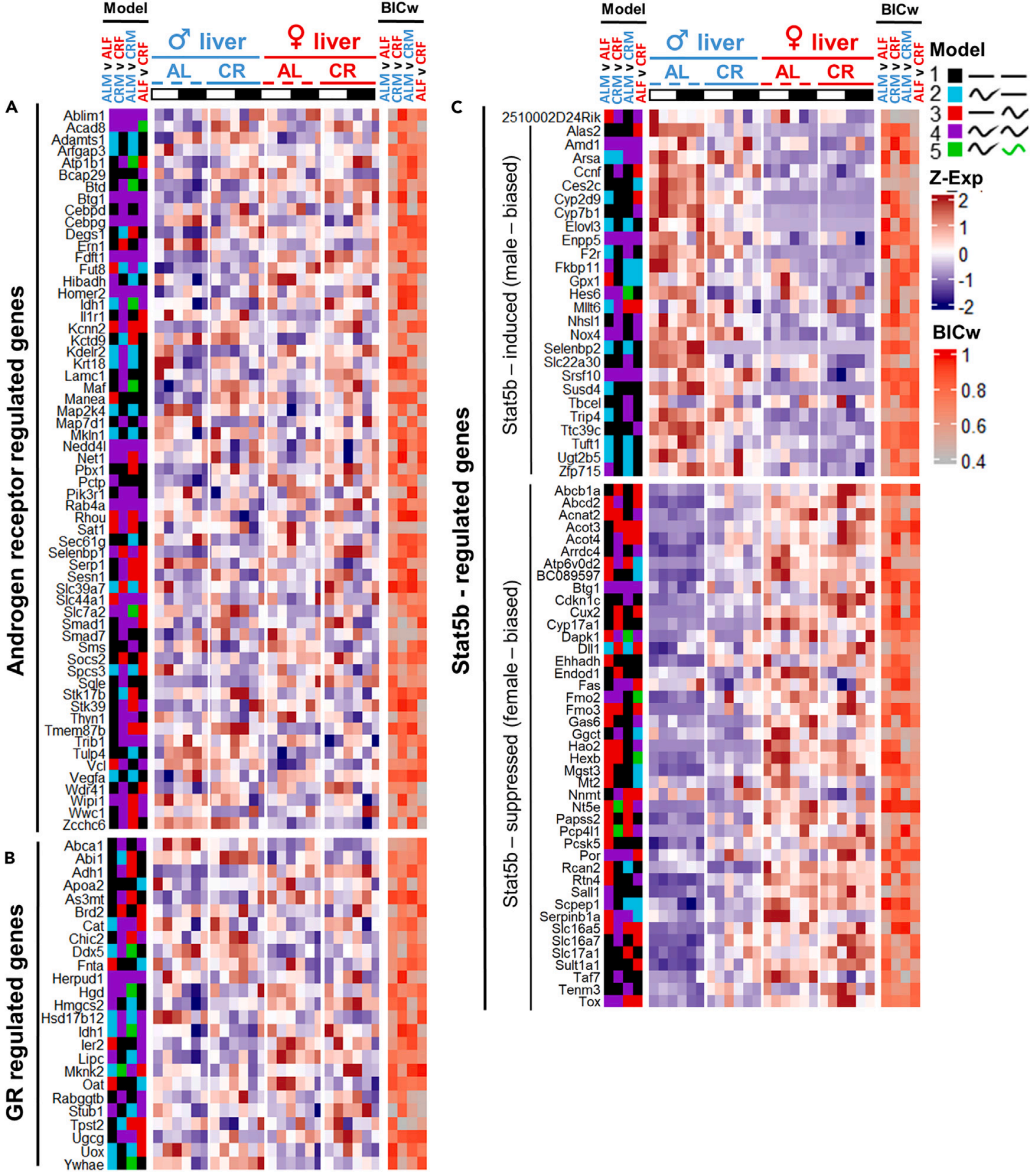
mRNA profiles of hormone-regulated genes The heatmaps show genes with rhythmic expression profiles in at least one experimental group. (A) Regulated by androgen receptor (Supplemental information of Jin et al.^66^). (B) Regulated by glucocorticoid receptor (Supplemental information of Phuc Le et al.^67^). (C) Regulated by GH-JAK-Stat5b axis (Supplemental information of Oshida et al.^68^). Data are represented as mean of *Z* scores (n = 3) per group, per time point. Annotation on the left side of the heatmap represents the model (category) to which the genes were assigned by CompareRhythms analysis using four pairwise comparisons, indicated on the top of the heatmap (AL male vs. AL female; CR male vs. CR female; AL male vs. CR female; AL female vs. CR female, see Tables S1 and S2). Model legend and color code is on the top right of the figure panel. Annotation on the right side of the heatmap represents BIC Schwartz weight value for each of the comparisons (see STAR Methods). Light-dark bars on top represent light and dark phase of the day. Figure color coding: Dash lines represent AL, solid lines – CR, blue – male, red – female liver. Order of the gene expression *Z* score averages within each group on the heatmap follows the time point order of the experimental design – ZT2,6,10,14,18,22. Order of the experimental groups on heatmaps is *ad libitum* male liver (♂AL); calorie-restricted male liver (♂CR); *ad libitum* female liver (♀AL); calorie-restricted female liver (♀CR) left to right, respectively.

Endocrine control of liver sexual dimorphism is well documented.^64^ We have mined the available data for steroid hormone receptors (AR and ER), growth hormone-dependent TF STAT5b, and glucocorticoid receptor (GR) targets^65^^,^^66^^,^^67^^,^^68^ (Figures 11 and 12; Table 1). We have found that the fraction of oscillating genes varied from 15% (ER-dependent genes in male liver under AL) to 47% (AR targets in male liver under CR). As expected, larger fraction of estrogen (E2)-sensitive and ER-dependent genes oscillated in female liver, while larger fraction of AR targets oscillated in males under AL condition (Table 1). Under AL males had higher fraction of oscillating targets for GR, while females had a higher fraction of oscillating STAT5b targets. Interestingly, CR increased oscillation in both sexes for all groups of endocrine targets with a slightly higher increase in AR- and ER-regulated genes (Table 1). GR targets showed stronger rhythmic response to CR in female liver (Table 1).

**Table 1:** Numbers and percentages of rhythmic genes among the hormone-regulated targets

Source	Gene set	AL male	CR male	AL female	CR female	Total
		№ (%)	№ (%)	№ (%)	№ (%)	№
Palierne et al. 2016	E2-sensitive	25 (20%)	37 (30%)	36 (29%)	44 (35%)	124
Palierne et al. 2016	ER-dependent basal	23 (15%)	41 (27%)	33 (22%)	45 (30%)	150
Jin et al. 2013	AR-regulated	45 (37%)	57 (47%)	36 (30%)	54 (44%)	122
Phuc Le et al. 2005	GR-regulated	16 (30%)	17 (32%)	14 (26%)	21 (40%)	53
Oshida et al. 2016	Stat5b-regulated	40 (27%)	48 (33%)	50 (34%)	55 (38%)	146

The table is showing the number of rhythmic genes and their percentages from total number of genes in each of the referenced gene sets and in each of the experimental groups. Gene set abbreviations: E2 -sensitive: genes that changed their expression level in wild-type female mouse liver upon 17β-estradiol treatment; ER-dependent: genes that were differentially expressed between livers of female wild-type and estrogen receptor-null mice^65^; AR-regulated: genes commonly reported to be regulated by androgen receptor (from the list of overlapping genes between 4 studies outlined in Jin et al.^66^); GR-regulated: overlapping genes which were both differentially expressed in male mouse liver upon dexamethasone injection and had their promoters occupied by glucocorticoid receptor assayed by ChIP-on-Chip.^67^ Stat5b-regulated: gene list that was derived from multiple comparisons between livers of wild-type male and female mice and between wild-type and STAT5b-null mice.^68^

## Discussion

Here, we have demonstrated sexual dimorphism in the liver transcriptome on two diets, as well as the difference in the response of circadian transcriptome to CR. Growing body of evidence demonstrates sexual dimorphism in various aspects of physiology. Mitchell et al. showed a differential sex-specific effect on metabolism and lifespan extension.^1^ Sex dimorphism in the transcriptome was also observed. Components of oxidative phosphorylation machinery, fatty acid biosynthesis enzymes, nucleosome components, and urinary proteins were differentially expressed between sexes under AL and CR and showed differential induction or suppression in response to CR at a single time point (the beginning of light phase when mice received food).^1^ We have observed similar changes in gene expression, and, in addition to these differences, we noted that many of the genes encoding oxidative phosphorylation components are predominantly rhythmic in female liver under AL diet, which suggests some differences may be accounted for by circadian rhythmicity (Figure S3). There is also a sexual dimorphism in response to acute fasting.^69^ Numerous genes involved in energy-producing pathways, fatty acid and triglyceride biosynthesis, as well as amino acid metabolism are differentially expressed between fasted male and female liver.^69^ We found that many of these genes demonstrated sexual dimorphism under CR and the effect was time of the day dependent.

Liver rhythmic transcriptome under AL diet was published by multiple groups, but the response of circadian transcriptome to various anti-aging feeding paradigms is a subject of ongoing research.^25^^,^^28^^,^^29^^,^^70^^,^^71^ Acosta-Rodriguez et al. reported a positive effect of timed CR on transcriptomic rhythmicity and lifespan extension in male mice,^28^ while Sato et al. reported increase in rhythmic transcripts, as well as CR-induced reprogramming of circadian liver transcriptome and the increase in rhythmicity in NAD+ metabolism genes on a mixed sample containing both male and female mice.^29^ Our comparisons between reports confirmed these findings and pointed to the instances where sex stratification may be necessary to detect rhythmic changes in gene expression, such as rare cases of rhythmic genes with large phase differences between sexes.

One of the major determinants of liver sexual dimorphism is growth hormone-dependent transcriptional factor STAT5b, as reported by single-time-point studies.^68^^,^^72^^,^^73^ Differential expression of steroid hormone receptors in the SCN and its afferents/efferents was also well described;^24^ therefore we could not rule out their potential contribution to the rhythmic sex dimorphism of the liver transcriptome via hypothalamic–pituitary–gonadal (HPG) and hypothalamic–pituitary–adrenal (HPA) axes. Circadian variation in serum testosterone (T) was previously noted in mice.^74^ Although with strain differences, the tendency toward higher circulating T levels during the light phase of the day was observed irrespective of whether the profile fits in circadian pattern or not.^74^ Strong circadian pattern in serum estradiol (E2) with a peak during end of the dark phase was reported for female rats.^75^ Serum corticosterone was reported to have circadian oscillation in rats in both sexes with a peak during transition from light to dark phase of the day; however the amplitude was 3 times larger in female rats.^76^ Indeed, we have outlined the contribution of hormone signaling to the dimorphism in rhythmicity (Table 1; Figures 11 and 12). While the reported targets constitute only a minor portion of rhythmic transcriptome, they may help to explain the differences in expression and/or amplitude of the most prominent gene candidates presented in this study. For instance, male-dominant *Elovl3* and *Hsd3b5* are STAT5b-induced genes. They lose rhythmicity under CR, and so do some of the other STAT5b targets (Table 1, Figure 12C). *Oat* is both ER-dependent and regulated by glucocorticoid signaling; its higher amplitude of expression in female liver may be potentially explained by female profiles of serum corticosterone and estradiol. *Atp6v0d2* is both ER-dependent and STAT5b-suppressed gene which explains its drastic female bias in expression (Figure 1D). Similarly, *Cux2* and *Hao2* as well as previously reported rhythmic *Fmo3*^33^ are *Stat5b*-suppressed genes which explains their strong female bias (Figures 4D and 7D), while male-rhythmic *Snhg11* and *Cth* are ER dependent (Figures 4D and 5M).

Recently, sex dimorphism was reported in circadian transcriptomes from human samples^32^ and in mice on high-fat diet challenged with chronic misalignment^31^ While the experimental paradigms and organisms were different, we were able to replicate the observation that larger fraction of rhythmic genes oscillated under AL in female liver than in male liver; the same was observed between male and female mice on high-fat diet provided AL.^31^ We also confirmed a similar phase distribution between male and female oscillating liver transcriptomes in mice compared against previously reported human data, as well as differential rhythmicity in flavin monooxygenases.^31^ More detailed comparison was possible with AL transcriptomic data from Weger et al.; we have independently verified sexually dimorphic and non-dimorphic targets and observed the similarity of physiological data (food intake and locomotor activity).^30^ Weger et al. also reported sex-specific differences in the expression of the genes involved in carbon folate and glutathione network.^30^ We observed sexual dimorphism in rhythmicity under AL in glutathione and nucleotide metabolism, while one carbon pathway was strongly rhythmic in both sexes with the exception of male-rhythmic *Cth* (Figure S2).

This study contributes to the understanding of sexual dimorphism dynamics; however, in order to understand the downstream physiological effect of sex dimorphism in rhythmic mRNA, it would strongly benefit from polar and non-polar metabolomics data follow-up for correlative comparison with the data from Figures 9, 10, S2, and S3, since we have observed strong sex-specific oscillations in a handful of biochemical enzymes, such as *Elovl3*, *Fasn*, *Acsl5*, and *Asns*. It would be interesting to see if protein expression and/or activity followed mRNA profiles. Candidates with the largest amplitudes can be assessed for protein expression; a comparison with proteomic circadian dataset needs to be considered since only a fraction of rhythmic proteins follows rhythmic mRNA profile.^77^ Ideally, building a prediction model of protein synthesis and/or metabolite dynamics from mRNA based on this and other datasets is a perspective future direction. Since sex-specific rhythmic genes were enriched in metabolic processes, like lipid metabolism, it is worth assessing potential sex differences in ketogenesis and lipid storage throughout the day between sexes, as well as DNA damage response and autophagy.

The upstream regulation of sexually dimorphic rhythmic transcription is outlined here briefly using predicted TF networks. The actual involvement of these TFs will also need to be confirmed using chromatin immunoprecipitation (ChIP) assays and/or transgenic animals and tissue-specific knockout models. The data on endocrine control of circadian gene expression can be reinforced by detection of serum levels of each respective hormone, preferably using repeated serum collections from the same subject animals over time.

In conclusion, we contribute to the growing body of evidence of sexual dimorphism in the liver and show that oscillating part of the liver transcriptome is different between sexes.

### Limitations of the study

The animals were not subjected to constant darkness; therefore the oscillating transcriptomic data reported here likely represent an output of the genes driven by both the light and the food. Studies with circadian mutants, extended constant-darkness protocols, and alternative feeding schedules are necessary to determine whether sexual dimorphism in rhythmicity is retained in solely clock-controlled or system-driven portions of the transcriptome. Additionally, even though mice were age-matched and samples were collected over 24-h period, we did not control for the phase of estrous cycle in female mice. Future investigations are warranted to delineate the regulation of sex-specific oscillations and sex-specific diet response of the liver rhythmic transcriptome.

## STAR★Methods

### Key resources table

**Table:** 

REAGENT or RESOURCE	SOURCE	IDENTIFIER
**Antibodies**		

rabbit-raised anti-GAPDH	Cell Signaling Technology	Cat# 5174S, lot 8; RRID: AB_10622025
rabbit-raised anti-pS235/236 S6	Cell Signaling Technology	Cat# 2211BC, lot 22; RRID: AB_331679
total ribosomal protein S6 mouse-raised monoclonal antibody	Santa Cruz	Cat# sc-74459, lot J1416; RRID: AB_1129205
Anti-rabbit IgG, HRP linked	Cell Signaling	Cat# 7074S; RRID: AB_2099233
Anti-mouse IgG, HRP linked	Cell Signaling	Cat# 7076S; RRID: AB_330924

**Critical commercial assays**		

RNeasy Mini Kit	QIAGEN	Ref #74104, Lot#163035346
NEBNext Ultra™ II RNA Library Prep Kit for Illumina	New England Biolabs	NEB #E7775
CVS Health Advanced Blood Glucose Meter	CVS	Item # 402723
CVS Health Glucose Meter Strips	CVS	Item # 260964

**Deposited data**		

RNA-seq Raw and Metadata from male mouse liver	Mezhnina et al.^70^	GSE211975
RNA-seq Raw and Metadata from female mouse liver	This paper	GSE216416
Raw and processed data from Figures 1, 2, 3, 4, 5, 6, 7, 8, 9, 10, 11, 12, and S1–S3	This paper	https://doi.org/10.17632/twd8f2zdz7
Raw data referenced in Figure 6 (between-study comparison)	Acosta-Rodriguez et al.^28^	GSE190939
Raw data referenced in Figure 6 (between-study comparison)	Sato et al.^29^	GSE93903
Processed data referenced in Figure 7 (between-study comparison)	Weger et al.^30^	GSE77221
Processed data referenced in Figure 7 (between-study comparison)	Talamanca et al.^32^	GTEx V8 (dbGaP Accession phs000424.v8.p2

**Biological Samples**		

Mouse Liver Tissue	This paper	N/A
Mouse Live-Collected Tail Vein Blood	This paper	N/A

**Experimental models: Organisms/strains**		

C57BL/6J mice	This paper	N/A

**Software and algorithms**		

ChEA3 – ChIP-X Enrichment Analysis	Keenan et al.^38^	Ver. 3
Bowtie	Langmead et al.^78^	Ver. 1.3.1
RSEM	Li & Dewey^79^	Ver. 1.2.3
EBSeq	Leng et al.^80^	N/A
CompareRhythms	Pelikan et al.^81^	N/A
JTK_Cycle	Hughes et al.^82^	N/A
MetaCycle	Wu et al.^83^	N/A
Complex Heatmap	Gu et al.^84^	N/A
R	R Core^85^	Ver. 4.3.0
Gene Ontology	Huang et al.^86^	DAVID 2021
Graph Pad Prism	Graph Pad Software	Ver. 6
Image Studio Lite	LI-COR Biosciences	Ver. 5.2
PAS Software	San Diego Instruments	N/A

**Instruments**		

Ohio Super Computer	Dell, Intel® Xeon®	Owens Cluster
Illumina NovaSeq 6000 platform	Novogene Corporation	NovaSeq 6000
Nano Drop 2000	Thermo Fisher Scientific	ND-2000
Bioanalyzer 2100	Agilent Technologies	N/A
Scientific Imaging film and Odyssey FC imaging system	LI-COR Biosciences	Odyssey FC
PAS Home Cage Photobeam System	San Diego Instruments	N/A

### Resource availability

#### Lead contact

Further information and requests for resources and reagents should be directed to and will be fulfilled by the lead contact, Dr. Roman Kondratov (r.kondratov@csuohio.edu).

#### Materials availability

This study did not generate new unique reagents.

#### Data and code availability

• The transcriptomic RNAseq data have been deposited and made publicly available via the Gene Expression Omnibus repository [GSE216416; GSE211975^70^] as of the date of publication. Processed RNA-seq data is available for quick access as Tables S1 and S2. Raw and processed data from the main text and supplemental figures of this article have been deposited at Mendeley Data repository [https://doi.org/10.17632/twd8f2zdz7]. This paper also analyzes existing, publicly available data. The accession numbers for the datasets are listed in the key resources table.
• This study did not generate original code.
• Any additional information required to reanalyze the data reported in this paper is available from the lead contact upon request.

### Experimental model and study participant details

#### Experimental animals

All the animal studies were performed with approval from the Institutional Animal Care and Use Committee (IACUC) of Cleveland State University (Protocol No. 21160-KON-S). The care and use of mice were carried out in accordance with the guidelines of the Institutional Animal Care and Use Committee (IACUC) of the Cleveland State University. C57BL/6J mice were maintained on 12 h light: 12 h dark cycle (LD12:12; light on at ZT0 and off at ZT12). Male and female mice used in the experiments were 12–15 weeks of age at the start of feeding protocol introduction. Animals were maintained in groups of three–four littermate animals per cage.

### Method details

#### Feeding protocols

Animals were fed 5008 LabDiet (proteins 26.5%, fat 16.9%, carbohydrates 56.5%). AL mice had around the clock access to food on the hopper of the cage. CR mice received the food on the floor of the cage once per day 2 h after the lights were turned off (ZT14). CR was started by gradual reduction of food amount provided – 1^st^ week – by 10%, 2^nd^ week – by 20%, 3^rd^ week – by 30% from 100% estimated AL intake. There was no restriction to water intake in both AL and CR groups. Food intake in ad-libitum animals was measured hourly for 3 days. Food intake in calorie restricted animals was measured every 15 minutes after the food was provided at zt14, since all of the food is consumed by calorie restricted animals in less than 2-hour window. Body weight of animals on both diets was monitored twice a week. At 5 months of age, liver tissues were collected across the day every 4 hours throughout regular LD12:12 cycle, immediately frozen and kept at −80°C for subsequent processing.

#### In-cage locomotor activity

In-cage locomotor activity was evaluated on single caged mice in their home cages using PAS Home Cage Photobeam System (San Diego Instruments). Locomotor activity was continuously monitored and automatically recorded every 60 minutes for 5 days. The data were analyzed using the PAS software.

#### Blood glucose measurement

Blood glucose measurements were done by tail vein puncture using CVS Health Advanced Blood Glucose Meter with CVS Health Advanced Glucose Meter Strips for 0.5 microliter sample.

#### Western blot analysis

Tissues were collected and frozen on dry ice. Frozen liver pieces were lysed in fresh cell signaling lysis buffer containing final concentrations of 0.02M Tris pH 7.5, 0.15M NaCl, 1mM EGTA, 1mM EDTA, 1% Triton-X, 2mM Na_2_P_2_O_7_, 1mM BGP, 0.2mM Na_2_VO_4_, 10mM NaF, 1mM PMSF. Final buffer mix contained 1% Protease (P8340) and 1% Phosphatase (P5726) Inhibitor Cocktail (Sigma-Aldrich, St. Louis, MO, USA). Lysis was performed immediately on ice by a series of short 1s pulses using sonicator (Fisher Scientific, Hampton, NH, USA). Protein concentration was determined on spectrophotometer by Bradford protein assay kit, Cat #5000006 (Bio-Rad Laboratories, Hercules, CA, USA) using Pierce™ Bovine Gamma Globulin Standard, Cat# 23212 (Thermo Scientific, Waltham, MA, USA) according to manufacturer’s protocol and lysates were stored at −80°C. Loading mix containing 0.1M Tris Cl pH6.8, 4% SDS, 25% Glycerol, 2.5% 2-Mercaptoethanol + a drop of bromophenol blue was used to correct remaining between-sample concentration differences. 45ug of protein was loaded on 4–12% bis-tris gels (Cat # NP0323BOX, ThermoFisher, Waltham, MA, USA) electrophoresed in a buffer containing 0.05M MOPS free acid, 0.05M Tris Base, 3.5 mM SDS, 1mM EDTA. Protein was transferred on PVDF membrane (Cat # 88518, Thermo Scientific, Waltham, MA, USA) in cold transfer buffer containing 0.025M Tris, 0.2M Glycine, 20% Methanol on ice. Quality of protein transfer was confirmed by Ponceau S staining (Cat # A40000279, Thermo Scientific, Waltham, MA, USA) of the membrane. Primary antibodies: rabbit-raised anti-GAPDH antibody 5174S, lot 8, 1:3000; P-S6 rabbit-raised antibody 2211BC, lot 22, 1:3500 (Cell Signaling, Danvers, MA); total ribosomal protein S6 mouse-raised monoclonal antibody sc-74459, lot J1416, 1:4500 (Santa Cruz, Dallas, TX). All of the primary antibodies were diluted in 5% BSA in proportions indicated in protocols by the respective manufacturers. Secondary antibodies used: Anti-rabbit IgG, HRP linked (Cell Signaling, 7074S, 1:5000) and Anti-mouse IgG, HRP linked (Cell Signaling, 7076S, 1:3000) dissolved in 1% milk in TBST. Blot images were made using Scientific Imaging film and Odyssey FC imaging system (LI-COR). Protein analysis and quantification was done using Image Studio Lite Version 5.2 software.

#### RNA isolation

Total RNA was isolated from mouse liver tissue using mini spin column QIAGEN RNeasy Mini Kit (Ref #74104, Lot#163035346, Hilden, Germany) according to the manufacturer’s protocol. Briefly, flash frozen animal tissues (30mg per sample) were placed in 600 μl of RNA lysis buffer (RLT - guanidine salt added by manufacturer + 20 μl of 2M DTT per 1ml of buffer RLT added right before RNA isolation) and immediately homogenized. Lysate was centrifuged for 3 min at 12000rpm, supernatant was thoroughly mixed with equal amount of 70% ice cold ethanol. 700 μl of sample was centrifuged for 15s at 8000g and flow-through was discarded. One washing step was performed using RNA wash buffer (RW1; 700 μl per sample; 15s, 8000g) and two steps with RNAse-free pellet enhancer buffer (4-fold diluted RPE concentrate in 99% ethanol; 500μl per sample; 15s, 2min, 8000g). RNA was eluted with 30 μl of RNAse free water (1min, 8000g) to the fresh 1.5 ml collection tube. All centrifugation steps were performed at room temperature (23°C). Sample purity was checked on Nano Drop (Thermo Fisher Scientific, Waltham, MA, USA) with 260/280 ratio >2.0 and 260/230 ratio >2.0 for every sample.

#### RNA-sequencing

The RNA sequencing was performed at Novogene Corporation Inc (Sacramento, CA, USA). RNA integrity was verified on 1% agarose gel, purity – on NanoPhotometer (IMPLEN, CA, USA), quantity and integrity - using RNA Nano 6000 Assay Kit of the Bioanalyzer 2100 (Agilent Technologies, CA, USA). 1 μg of RNA per sample was used as an input and sequencing library was prepared using NEBNext Ultra™ II RNA Library Prep Kit for Illumina (New England BioLabs, USA). mRNA was poly-A selected from total RNA using poly-T oligo-attached magnetic beads. Fragmentation was performed using divalent cations under high temperature in NEBNext First Strand Synthesis Reaction Buffer (5X). First strand cDNA was synthesized using random hexamer primer and M-MuLV Reverse Transcriptase (RNase H). Second strand cDNA synthesis was subsequently performed using DNA Polymerase I and RNase H. Remaining overhangs were converted into blunt ends by exonuclease/polymerase reaction. After adenylation of 3’ ends of DNA fragments, NEBNext Adaptor with hairpin loop structure were ligated to prepare for hybridization. The library fragments were purified with AMPure XP system (Beckman Coulter, Beverly, USA) in order to select cDNA fragments of preferentially 150∼200 bp in length. 3 μl USER Enzyme (NEB, USA) was incubated with size-selected, adaptor ligated cDNA at 37 °C for 15 min followed by 5 min at 95 °C before PCR. Then PCR was performed with Phusion High-Fidelity DNA polymerase, Universal PCR primers and Index (X) Primer. PCR products were purified using AMPure XP system. Library quality was assessed on the Agilent Bioanalyzer 2100. The clustering of the index-coded samples was performed on a cBot Cluster Generation System using PE Cluster Kit cBot-HS (Illumina) according to the manufacturer’s instructions. After cluster generation, the library preparations were sequenced on an Illumina NovaSeq 6000 platform and 50M paired-end reads (∼150bp) per sample were generated.

Subsequent processing was performed in-house using connection to Ohio Supercomputer Owens Cluster Server (Columbus, OH, USA). RNA-seq reads were mapped to the mouse protein coding genes (Ensembl: Mus_musculus; GRCm38 (mm10)) using Bowtie^78^ allowing up to 2-mismatches. The gene expected read counts and Transcripts Per Million (TPM) were estimated by RSEM (v1.2.3).^79^ The TPMs were further normalized by EBSeq^80^ R package to correct potential batch effect. The transcriptomics data are available via the Gene Expression Omnibus Repository (GEO): GSE211975 for the samples from male liver and GSE216416 for the samples from female liver (refer to key resources table). Transcriptomic data from all the samples is also summarized in Table S1 (n=3 per time point per group, with the exception of female ad-libitum ZT22 time point (n=2)).

### Quantification and statistical analysis

#### Analysis of rhythmicity

Analysis of transcript rhythmicity was performed using model selection approach of CompareRhythms R package (Bharath Ananthasubramaniam Lab, Berlin, Germany).^81^ Normalized TPMs were used as an input and BIC Schwartz weight cutoff was set at 0.4 with a period of 24 hours and rhythmicity/comparison FDR at 0.05 after the truncation of data at normTPM>1. Output of CompareRhythms provides phase value in radians, therefore phase was calculated by multiplying the indicated phase value by period, divided by (2π), in this study: phase = (phase in radians ∗ 24h)/(2π). R software was used with the code available from (https://github.com/bharathananth/compareRhythms). Pairwise CompareRhythms analyses were conducted between the indicated pairs of data groups in Figures 2A, 4A, 5A, 5F, 6A, 6F, 9, 10, 11, and 12. Venn diagram comparisons were used in Figure 6L, M (based on independent JTK_CYCLE analysis^82^^,^^83^); in Figures 5B, 5G, 7A, 8A, and 8B (based on the results from independent pairwise CompareRhythms analysis). No statistical analysis was performed between data compared by Venn diagram method. For Figures 6L and 6M - JTK_CYCLE via MetaCycle package (Hughes Lab, Toronto, ON, Canada)^82^^,^^83^ was selected due to platform differences (Illumina RNA-Seq vs Affymetrix Microarray) and to match the method used in the published data for comparison.^28^^,^^29^ Differential rhythmicity comparison in Figure 7 was performed by overlapping the list of genes in each category assigned by model selection between AL male and AL female samples independently in this study and Weger et al. 2019.^30^ Genes were assigned into categories by similar model selection BIC approach (as described in^30^^,^^81^) with per=24h, BIC Schwartz weight>0.4, FDR=0.05, but the transcriptomic data was processed and normalized separately in each study. Venn-diagram contrasts in Figures 8A and 8B originate from AL vs CR pairwise CompareRhythms comparison in each sex. Four pairwise CompareRhythms analyses were conducted to confirm sex and diet effects on rhythmicity in 5M, 6E,K,J: AL male vs AL female, CR male vs CR female, AL male vs CR male, AL female vs CR female. In Figures 8, 9, 10, 11, and 12 - left side heat map annotations represent the assigned models for each of the four CompareRhythms pairwise comparisons; while the right side annotations represent the BICW. Heat map for rhythmic transcripts was generated using Complex Heatmap package^84^ in Rstudio (https://www.rstudio.com/).^85^

#### Biological process and gene ontology enrichment analysis

The Database for Annotation, Visualization, and Integrated Discovery (DAVID) Ver. 2021 (https://david.ncifcrf.gov/)^86^^,^^87^ was used for BP and GO analysis. Official gene symbols (common gene names) were used for the input for each subset of genes analyzed. Top 10 significantly enriched terms were presented for each analyzed subset of genes after removal of redundant BPs and GOs, negative log of p-value with Benjamini-Hochberg correction was used for ranking BPs and GOs, and number of genes in each BP and GO term was plotted in figures as bar graphs.

#### ChEA3 transcription factor enrichment analysis

Transcription Factor Enrichment Analysis was performed using ChEA3 – ChIP-X Enrichment Analysis (Ver. 3) tool (https://maayanlab.cloud/chea3/).^38^ Top 10 significantly enriched TFs were displayed for each group of genes discussed in the manuscript using “Mean Rank” option. The results were presented as bar graphs.

#### Analysis of variance

For timed and repeated measurements two-way ANOVA analysis was performed using GraphPad Prism, Ver 6. (Graph Pad Software, Boston, MA), with p-value < 0.05 as a cutoff for significance. For assessment of differences in total daily locomotor activity and food intake 2-tailed T-test was used with p-value <0.05 as a cutoff for significance.

## Supplemental information

Document S1. Figures S1–S3

Table S1. RNA-seq data table, related to Figure 2,4,5–12 RNA-seq data table containing counts, transcripts per million (TPMs) and normalized transcripts per million (nTPMs) for each gene and sample.

Table S2. Results of CompareRhythms analysis of RNA-seq data, related to Figure 2,4,5–12 Output from CompareRhythms analysis containing four pairwise comparisons: AL male vs AL female, CR male vs CR female, AL male vs CR male, AL female vs CR female.
