# Supplementary material for: Sexual dimorphism of circadian liver transcriptome
Source: iScience. 2024 Mar 12;27(4):109483. doi: 10.1016/j.isci.2024.109483 (PMC10973666; doi:10.1016/j.isci.2024.109483)
Supplement: Document S1. Figures S1–S3 [file mmc1.pdf]

iScience, Volume *27*

Supplemental information

Sexual dimorphism of circadian liver transcriptome

Artem A. Astafev, Volha Mezhnina, Allan Poe, Peng Jiang, and Roman V. Kondratov

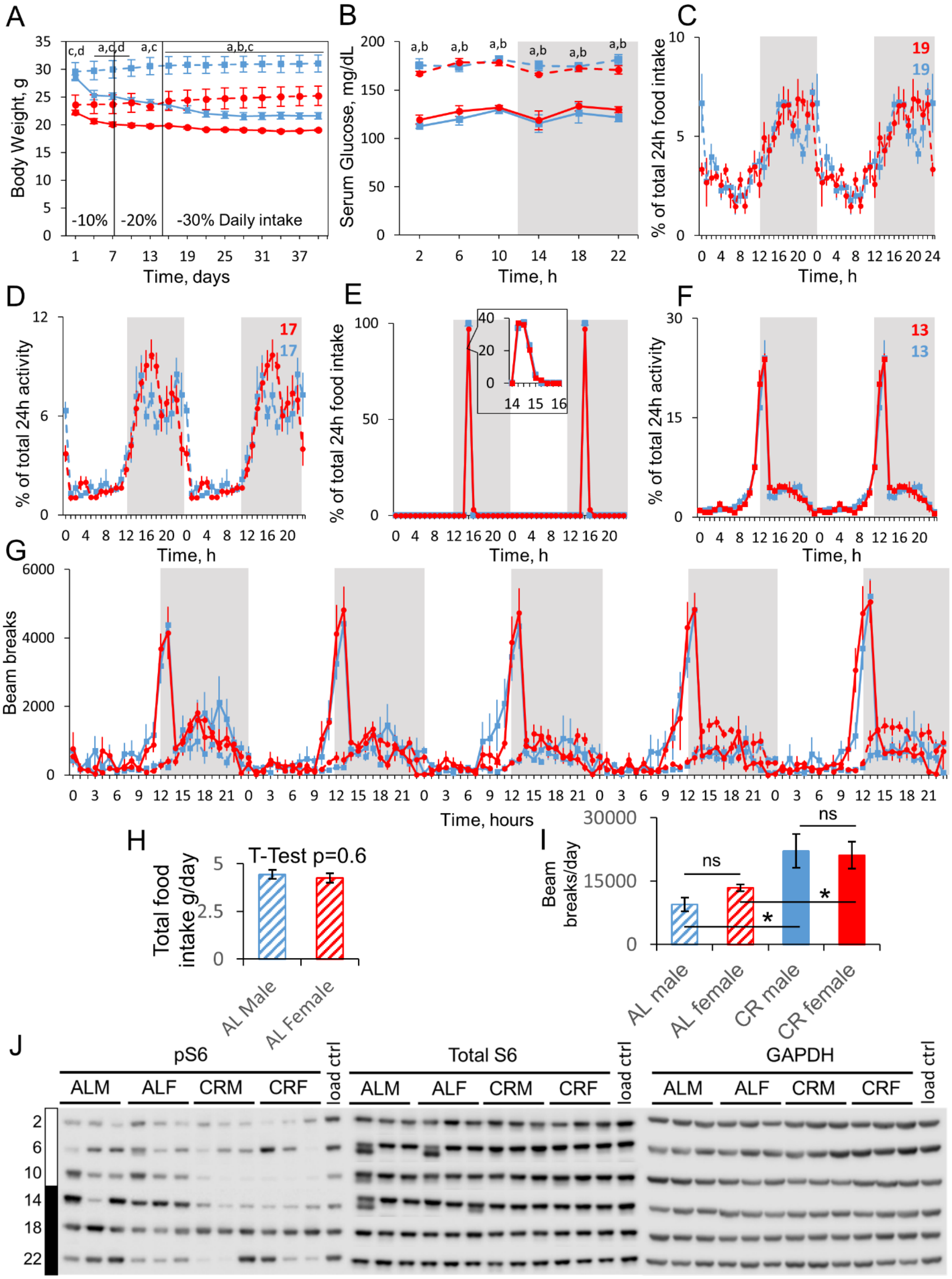
A
Body Weight, g
c,d
a,c,d
a,c
a,b,c
-10%
-20%
-30% Daily intake
Time, days
B
Serum Glucose, mg/dL
a,b
Time, h
C
% of total 24h food intake
19
19
Time, h
D
% of total 24h activity
17
17
Time, h
E
% of total 24h food intake
14 15 16
Time, h
F
% of total 24h activity
13
13
Time, h
G
Beam breaks
Time, hours
H
Total food intake g/day
T-Test p=0.6
AL Male
AL Female
I
Beam breaks/day
ns
ns
*
*
AL male
AL female
CR male
CR female
J
pS6
Total S6
GAPDH
ALM
ALF
CRM
CRF
load ctrl
2
6
10
14
18
22

Figure S1. Physiological parameters of experimental animals, Related to Figure 1 and Figure 3.

A. Time course of mouse bodyweight change in grams. Blue lines represent male mice, red – female mice. Data are presented as mean±SEM. Blue dashed line squares – AL males (n=3±SEM), blue solid line squares – CR males(n=3±SEM), red dashed line circles – AL females(n=3±SEM), red solid line circles – CR females(n=3±SEM). B. Serum glucose in milligrams per deciliter measured by tail vein puncture in live mice (mean, n=3±SEM) for all groups. Color code as specified in A. Significant differences between groups by two-way ANOVA are indicated as $p<0.05$: [a) AL male vs CR male; b) AL female vs CR female; c) AL male vs AL female; d) CR male vs CR female]. C. Food intake of AL mice represented as % to daily total intake, measured hourly for 3 days (n=5 ± SEM per group). D. Locomotor activity of AL mice represented as % to daily total activity. recorded hourly for 5 days (n=6 ± SEM per group). E. Food intake of CR mice represented as % to the daily total intake measured hourly + measured every 15 minutes for 3 days (n=3 ± SD per group). F. Locomotor activity in CR mice represented as % of total daily activity, recorded hourly for 5 days (n=7 for males and n=5 for females ± SEM per group). G. Continuous raw locomotion represented as mean of beam breaks±SEM, measured over 5 days. Line labeling as specified in A. H. Total daily food intake in AL mice – mean of 3 days (n=5 ± SEM per group). I. Total daily locomotor activity in AL mice – mean of 5 days (n=7 for males, 5 – for females ± SEM per group). Significant differences between groups by two-way ANOVA are indicated as *$p<0.05$. J. Quantification western blots of ribosomal protein S6 phosphorylated at p235/236, total protein S6 and control protein GAPDH. Color coding: blue dash – male AL; red dash – female AL; solid blue – male CR; solid red – female CR.

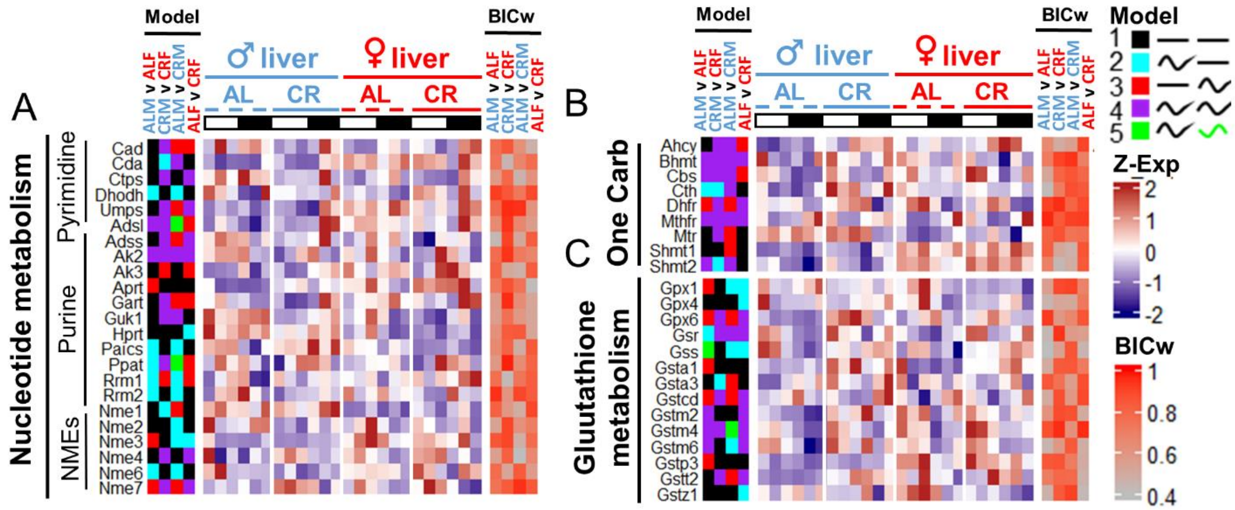

Figure S2. mRNA profiles of the genes involved in nucleotide metabolism and antioxidant defense, Related to Figure 7.

Heat maps of RNA-seq expression profiles of the genes involved in A. Nucleotide metabolism. B. One Carbon Folate metabolism. C. Glutathione metabolism. Data are represented as Z-scores (n=3) per group per time point. Annotation on the left side of the heat map represents the model (category) to which the genes were assigned by CompareRhythms analysis in each pairwise comparison indicated on top of the heatmap. Annotation on the right side of the heat map represents BIC Schwartz weight value (see methods). Light-dark bars on top represent light and dark phase of the day. Figure color coding: Dash lines represent AL, solid lines – CR, blue – male, red – female liver. Order of the gene expression Z-score averages within each group on the heat map follows the time point order of the experimental design – ZT2,6,10,14,18,22. Order of the experimental groups on heat maps is Ad-libitum male liver (♂AL); Calorie-restricted male liver (♂CR); Ad-libitum female liver (♀AL); Calorie-restricted female liver (♀CR) left to right respectively.

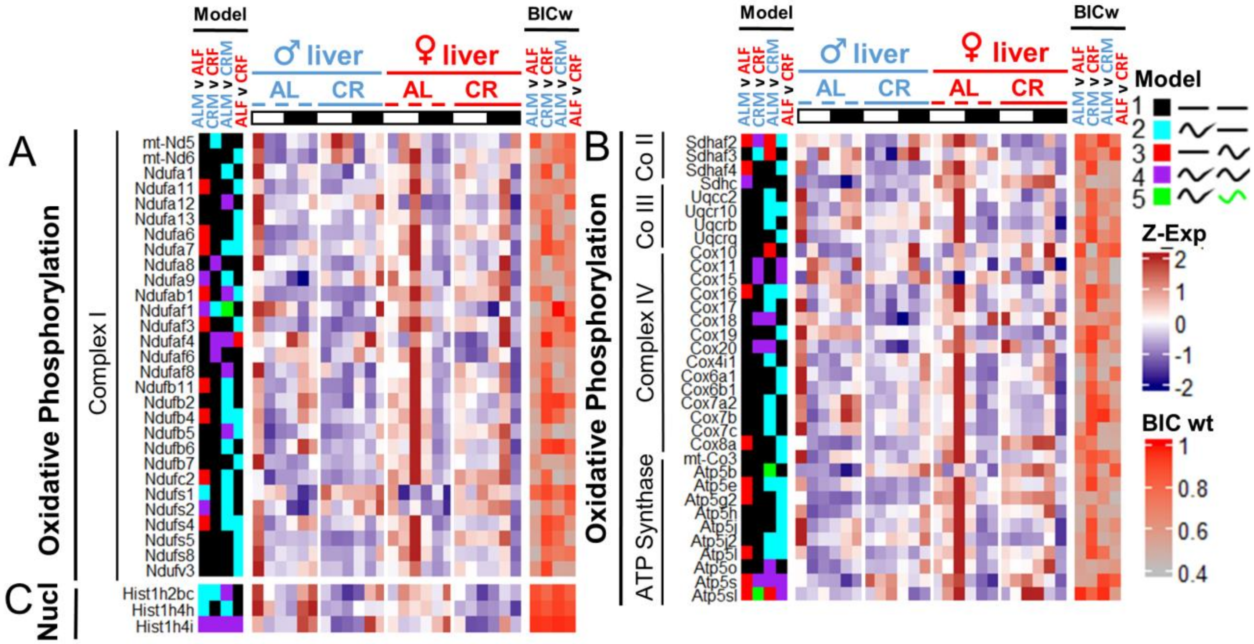

Figure S3. mRNA profiles of the genes that are predominantly rhythmic in AL female liver and lose rhythmicity under CR, Related to Figure 9.

Heat maps of RNA-seq expression profiles of the genes involved in A. Oxidative phosphorylation Complex I, B. Complex II-V, C. Components of nucleosome. Data are represented as Z-scores (n=3) per group per time point. Annotation on the left side of the heat map represents the model (category) to which the genes were assigned by CompareRhythms analysis in each pairwise comparison indicated on top of the heatmap. Annotation on the right side of the heat map represents BIC Schwartz weight value (see methods). Light-dark bars on top represent light and dark phase of the day. Figure color coding: Dash lines represent AL, solid lines – CR, blue – male, red – female liver. Order of the gene expression Z-score averages within each group on the heat map follows the time point order of the experimental design – ZT2,6,10,14,18,22. Order of the experimental groups on heat maps is Ad-libitum male liver (♂AL); Calorie-restricted male liver (♂CR); Ad-libitum female liver (♀AL); Calorie-restricted female liver (♀CR) left to right respectively.
